# The Role of Astrocytes in Synapse Loss in Alzheimer's Disease: A Systematic Review

**DOI:** 10.3389/fncel.2022.899251

**Published:** 2022-06-16

**Authors:** Lianne A. Hulshof, Danny van Nuijs, Elly M. Hol, Jinte Middeldorp

**Affiliations:** ^1^Department of Translational Neuroscience, University Medical Center Utrecht Brain Center, Utrecht University, Utrecht, Netherlands; ^2^Department Neurobiology and Aging, Biomedical Primate Research Centre, Rijswijk, Netherlands

**Keywords:** Alzheimer's disease, astrocyte, synapse, dementia, synapse loss, systematic review

## Abstract

Alzheimer's disease (AD) is the most common cause of dementia, affecting 35 million people worldwide. One pathological feature of progressing AD is the loss of synapses. This is the strongest correlate of cognitive decline. Astrocytes, as an essential part of the tripartite synapse, play a role in synapse formation, maintenance, and elimination. During AD, astrocytes get a reactive phenotype with an altered gene expression profile and changed function compared to healthy astrocytes. This process likely affects their interaction with synapses. This systematic review aims to provide an overview of the scientific literature including information on how astrocytes affect synapse formation and elimination in the brain of AD patients and in animal models of the disease. We review molecular and cellular changes in AD astrocytes and conclude that these predominantly result in lower synapse numbers, indicative of decreased synapse support or even synaptotoxicity, or increased elimination, resulting in synapse loss, and consequential cognitive decline, as associated with AD. Preventing AD induced changes in astrocytes might therefore be a potential therapeutic target for dementia.

**Systematic Review Registration:** https://www.crd.york.ac.uk/prospero/display_record.php?RecordID=148278, identifier [CRD148278].

## Graphical Abstract

AD astrocytes decrease the number of synapses *via* a variety of pathways. We distinguished a direct and indirect effect of astrocytes on synapse number. Direct: phagocytic capacity is impaired, affected by astrocyte expression of MERTK, MEGF10, C3, and ApoE4. Indirect: impaired glutamate transport & signaling, impaired Ca2+ signaling, and decreased TSP1 expression all can lead to a synaptotoxic environment, which indirectly reduces synapse number. Impaired glutamate transport is caused by a decrease in GLT1 and GLAST expression and leads to an increase in glutamate in the synaptic cleft, resulting in impaired NMDAR activity and excitotoxicity. Besides affecting phagocytosis, ApoE4 also increases inflammation, and Tau and Aβ aggregation, thereby further promoting synapse loss. The astrocyte (green) interacts with a synapse on a neurite (blue). The decrease in synapse number is visualized by more transparent synapses. 
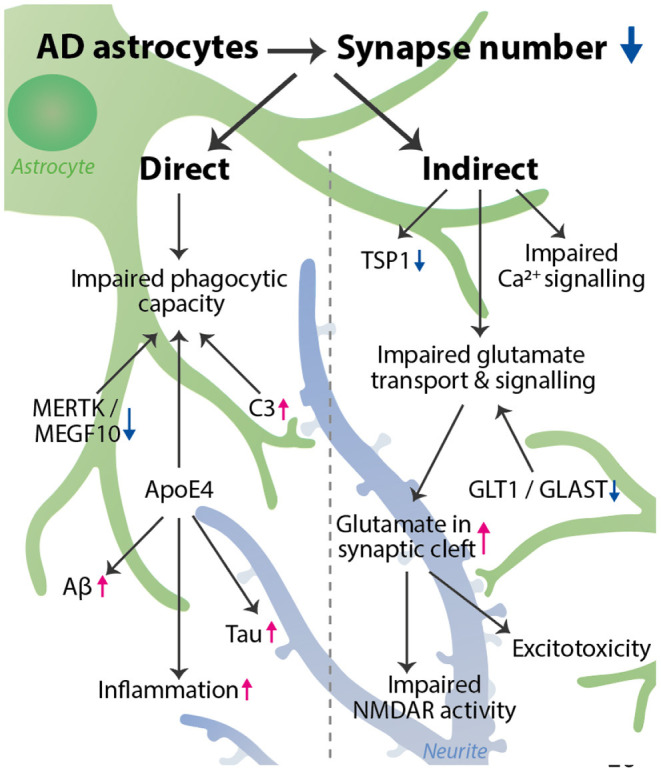

## Introduction

Alzheimer's disease (AD) is the most common cause of dementia, and since age is the major risk factor for this disease, the number of affected people will only increase in our aging population. There is an increasing amount of knowledge on the underlying genetic, cellular, and molecular processes that cause this disease, but this has so far not led to an effective drug to treat AD. One pathological feature of progressing AD is the loss of synapses, which is the strongest correlate of cognitive decline (Davies et al., 1987; DeKosky and Scheff, 1990; Terry et al., 1991; Wilde et al., 2016). Research has long focused on neurons and their molecular processes when studying AD and related synapse loss. However, it has become increasingly clear that glial cells, including microglia and astrocytes, play a crucial role in synapse formation, maintenance, and elimination.

Three distinct pathological hallmarks of AD are the development of amyloid-β (Aβ) plaques, tau tangles, and reactive gliosis. Aβ plaques are caused by an accumulation of Aβ-peptides, a cleavage product of amyloid precursor protein (APP) (reviewed in Haass and Selkoe, 1993; Selkoe and Hardy, 2016). Familial AD is caused by mutations, either in the APP gene or in the Presenilin genes (PS1 and PS2), which are essential components of gamma-secretase that cleaves APP (Citron et al., 1998; Cacquevel et al., 2012). These mutations, which ultimately lead to an accumulation of the Aβ protein, are used to generate mouse models of amyloidosis to study this aspect of the disease. However, another major hallmark of AD is the presence of tau tangles, which consist of aggregates of hyperphosphorylated tau protein in neurons, which leads to destabilization of microtubules (Oddo et al., 2003). Like amyloid plaques, tangles do not naturally form in rodents, also not in response to APP, PS1 or PS2 mutations, so to model this aspect of the disease, tau mutations are introduced in mice. In response to amyloid accumulation and hyperphosphorylation of tau, synapses and neurons are lost (Köpke et al., 1993; Alonso et al., 1994; Iqbal et al., 2005), and glial cells become reactive. Reactive gliosis is clearly present in AD patients and AD mouse models. Microglia become immune-activated and invade the Abeta plaques, and astrocytes respond by changes in their morphology and gene expression profile, resulting in a changed function compared to healthy astrocytes. For instance, increased expression of the intermediate filament protein glial fibrillary acidic protein (GFAP) is a commonly acknowledged marker for astrocyte reactivity (Eng et al., 1971; Eng and Ghirnikar, 1994; Osborn et al., 2016; Smit et al., 2021).

Most AD patients do not carry APP, PS1 or PS2 mutations leading to these pathological hallmarks, but many other risk factors can increase the chance of getting this disease. These risk factors are both non-genetic, such as increasing age, and genetic, such as the E4 variant of the Apolipoprotein E (ApoE) gene, which is present in more than half of all AD patients (Corder et al., 1993; Morikawa et al., 2005; Lambert et al., 2013; Jansen et al., 2019; Reiman et al., 2020; Wightman et al., 2021). When studying the role of astrocytes in AD, the ApoE genotype should also be considered as an important factor, since astrocytes are the main source for ApoE production in the brain (Xu et al., 2006) and the ApoE genotype causes functional changes in astrocytes (Verkerke et al., 2021).

Although the exact number of interactions is not entirely clear, it is predicted that in the human brain, astrocytes can contact up to 2 million synapses (Oberheim et al., 2006, 2009; Allen and Eroglu, 2017). The interaction between astrocytes and the pre- and postsynaptic membrane, also known as the tripartite synapse, ensures a proper environment for maintenance of synapses and efficient synaptic transmission. Astrocytes provide structural support for synapses, recycle and release neurotransmitters, maintain ion and water homeostasis, and express proteins that stimulate synapse formation (Allen and Eroglu, 2017). *In vitro* experiments with mouse hippocampal slice cultures show that direct contact of astrocytes with neurons is essential for proper synapse formation, and dendritic spines that are in contact with astrocytes live longer and have a more mature morphology compared to spines without astrocyte contact (Nishida and Okabe, 2007). Astrocytes aid in synapse formation both through providing structural support *via* cell adhesion and extracellular matrix molecules, and through expression and secretion of proteins that directly affect synapse formation. Examples of proteins expressed by astrocytes that are involved in synapse formation are thrombospondins, neurexins, and ephrins (more extensively reviewed in Allen and Eroglu, 2017; Hillen et al., 2018).

Astrocytes can also actively eliminate synapses through phagocytosis (Chung et al., 2013). Especially during development, there is an excess in the number of synapses being generated and selective elimination of aberrant synapses is an essential process of brain development (Neniskyte and Gross, 2017). Also in adulthood synaptic pruning occurs for the removal of inefficient or unnecessary synapses, which is important for synaptic plasticity and memory formation (Lichtman and Colman, 2000). As part of the innate immune system, the complement system is involved in synaptic pruning (Stevens et al., 2007; Luchena et al., 2018). The classical complement pathway is usually activated when a ligand binds to complement component 1q (C1q). Downstream complement component 3 (C3) is cleaved and can then binds to complement receptor 3 (CR3) in microglia, promoting phagocytosis. Although healthy astrocytes only express low levels of these complement system proteins, they induce the expression of C1q in neurons (Stevens et al., 2007; Luchena et al., 2018).

As astrocytes play a key role in synapse formation, functioning and elimination, a role for astrocytes in synapse loss in AD cannot be ignored. Previous research has highlighted the importance of astrocytes in AD-related neuronal and synaptic loss. Already in 1995, a correlation was found between a decrease in synapse density and an increase in the number of GFAP-positive astrocytes in both the frontal lobe and parietal lobe of AD patients, suggesting a role for astrocytes in synapse elimination (Brun et al., 1995). Other studies showed a decreased co-localization of astrocytes with synapses in the 5xFAD mouse model for AD, suggesting that proper interaction between astrocytes and synapses is essential for preserving synapses (Choi et al., 2021).

To provide an overview of the literature on the role of astrocytes in synapse loss in AD we designed a systematic search string centered around the terms “Alzheimer's Disease,” “Astrocyte” and “Synapse,” and incorporated both human and animal studies, *in vitro* and *in vivo*. We were surprised that only a very limited number of them discusses changes in synaptic protein expression or synapse number as an outcome measure. Though many papers discussed changes in synapse function, we chose to focus our review on synapse loss, which is the strongest correlate of cognitive decline. Therefore, this systematic review will give an overview of the current knowledge regarding underlying molecular mechanisms of how astrocytes can affect synapse number in AD, and by doing this contribute to cognitive decline.

## Methods

The main question for this systematic review is “What is the role of astrocytes and astrocyte reactivity on synapse number in Alzheimer's Disease?”. From this question we derived three keywords: “Alzheimer's Disease,” “Astrocytes,” and “Synapses.” For each keyword we made a systematic search term by combining their MeSH term with all relevant entry terms. The relevance of an entry term was determined by whether removing the term from the search string affected the number of hits. For the search string “Alzheimer's disease” we also added names of the most commonly used AD mouse models (source: https://www.alzforum.org/research-models/Alzheimer's-Disease/commonly-used). We then combined the search terms from the three keywords to make one systematic search string, which we fitted for both Pubmed and EMBASE.

The Pubmed search string is as follows:

*((“Alzheimer disease”[MeSH] OR “Alzheimer's Disease” OR “Alzheimer's Disease” OR “Alzheimer's” OR “Alzheimers” OR “Alzheimer” OR “Senile Dementia” OR “Presenile Dementia” OR “APP/PS1” OR “APPPS1” OR “APPxPS1” OR “Tg(APPswe,PSEN1dE9)85Dbo” OR “5xFAD” OR “Tg6799” OR “ APPNL-G-F/NL-G-F” OR “AppNL-G-F” OR “ARTE10” OR “TgCRND8” OR “KM670/671NL” OR “TASTPM” OR “TAS/TPM” OR “APPNL-F/NL-F” OR “PS/APP” OR “ PSAPP” OR “PS1(M146L)” OR “APP23” OR “B6-Tg/Thy1APP23Sdz” OR “PDAPP” OR “PD-APP” OR “3xTg” OR “3xTg-AD” OR “LaFerla” OR “APPswe/PSEN1dE9” OR “APdE9” OR “Borchelt” OR “rTg9191” OR “APPNLI” OR “Tg2576/Tau(P301L)” OR “APP(V717I)” OR “APPlon” OR “APP-london” OR “APP.V717I” OR “Tg2576” OR “App-Swe” OR “App-Sw” OR “AppSwe” OR “APP(sw)” OR “TAS10” OR “Tg(APPSw)” OR “rTgTauEC” OR “TauRD*ΔK280” OR “TauRD” OR “APP(OSK)-Tg” OR “APPOSK” OR “hTau.P301S” OR “hTau.P301S” OR “Tau P301L” OR “JNPL3”) AND (Astrocytes[Mesh] OR astrocytes OR Astrocyte OR Astroglia OR Astroglias OR Astro^*^ OR Glia OR “glia cell” OR “glial cell”)) AND (synapses[MeSH] OR “dendritic spines”[Mesh] OR synapse OR synapses OR “neuronal gap junctions” OR “axon terminals” OR “axon terminal” OR “mossy fibers” OR “mossy fiber” OR synaptic OR presynaptic OR postsynaptic OR synap^*^ OR PSD OR synaptosome OR spine OR spines OR spine^*^ *OR dendritic)*.

The EMBASE search string is as follows:

*((“azheimer's disease” OR “azheimer's disease”:ti,ab,kw OR “azheimer's disease”:ti,ab,kw OR alzheimers) AND ti,ab,kw OR alzheimer:ti,ab,kw OR “senile dementia”:ti,ab,kw OR “presenile dementia”:ti,ab,kw OR “app/ps1”:ti,ab,kw OR “appps1”:ti,ab,kw OR “appxps1”:ti,ab,kw OR “tg(appswe,psen1de9)85dbo”:ti,ab,kw OR “5xfad”:ti,ab,kw OR “tg6799”:ti,ab,kw OR “appnl-g-f/nl-g-f”:ti,ab,kw OR “appnl-g-f”:ti,ab,kw OR “arte10”:ti,ab,kw OR “tgcrnd8”:ti,ab,kw OR “km670/671nl”:ti,ab,kw OR “tastpm”:ti,ab,kw OR “tas/tpm”:ti,ab,kw OR “appnl-f/nl-f”:ti,ab,kw OR “ps/app”:ti,ab,kw OR “psapp”:ti,ab,kw OR “ps1(m146l)”:ti,ab,kw OR “app23”:ti,ab,kw OR “b6-tg/thy1app23sdz”:ti,ab,kw OR “pdapp”:ti,ab,kw OR “pd-app”:ti,ab,kw OR “3xtg”:ti,ab,kw OR “3xtg-ad”:ti,ab,kw OR “laferla”:ti,ab,kw OR “appswe/psen1de9”:ti,ab,kw OR “apde9”:ti,ab,kw OR “borchelt”:ti,ab,kw OR “rtg9191”:ti,ab,kw OR “appnli”:ti,ab,kw OR “tg2576/tau(p301l)”:ti,ab,kw OR “app(v717i)”:ti,ab,kw OR “applon”:ti,ab,kw OR “app-london”:ti,ab,kw OR “app.v717i”:ti,ab,kw OR “tg2576”:ti,ab,kw OR “app-swe”:ti,ab,kw OR “app-sw”:ti,ab,kw OR “appswe”:ti,ab,kw OR “app(sw)”:ti,ab,kw OR “tas10”:ti,ab,kw OR “tg(appsw)”:ti,ab,kw OR “rtgtauec”:ti,ab,kw OR “taurd*δk280”:ti,ab,kw OR “taurd”:ti,ab,kw OR “app(osk)-tg”:ti,ab,kw OR “apposk”:ti,ab,kw OR “htau.p301s”:ti,ab,kw OR “tau p301l”:ti,ab,kw OR “jnpl3”:ti,ab,kw) AND (“synapse”/exp OR “dendritic spine”/exp OR synapse OR synapses OR “neuronal gap junctions” OR “axon terminals” OR “axon terminal” OR “mossy fibers” OR “mossy fiber” OR synaptic OR presynaptic OR postsynaptic OR synap^*^OR psd OR synaptosome OR spine OR spines OR spine^*^ OR dendritic) AND (“astrocyte”/exp OR astrocyte:ti,ab,kw OR astrocytes:ti,ab,kw OR astroglia:ti,ab,kw OR astroglias:ti,ab,kw OR astro^*^:ti,ab,kw OR glia:ti,ab,kw OR “glia cell”:ti,ab,kw OR “glial cell”:ti,ab,kw).

**Figure 1:**
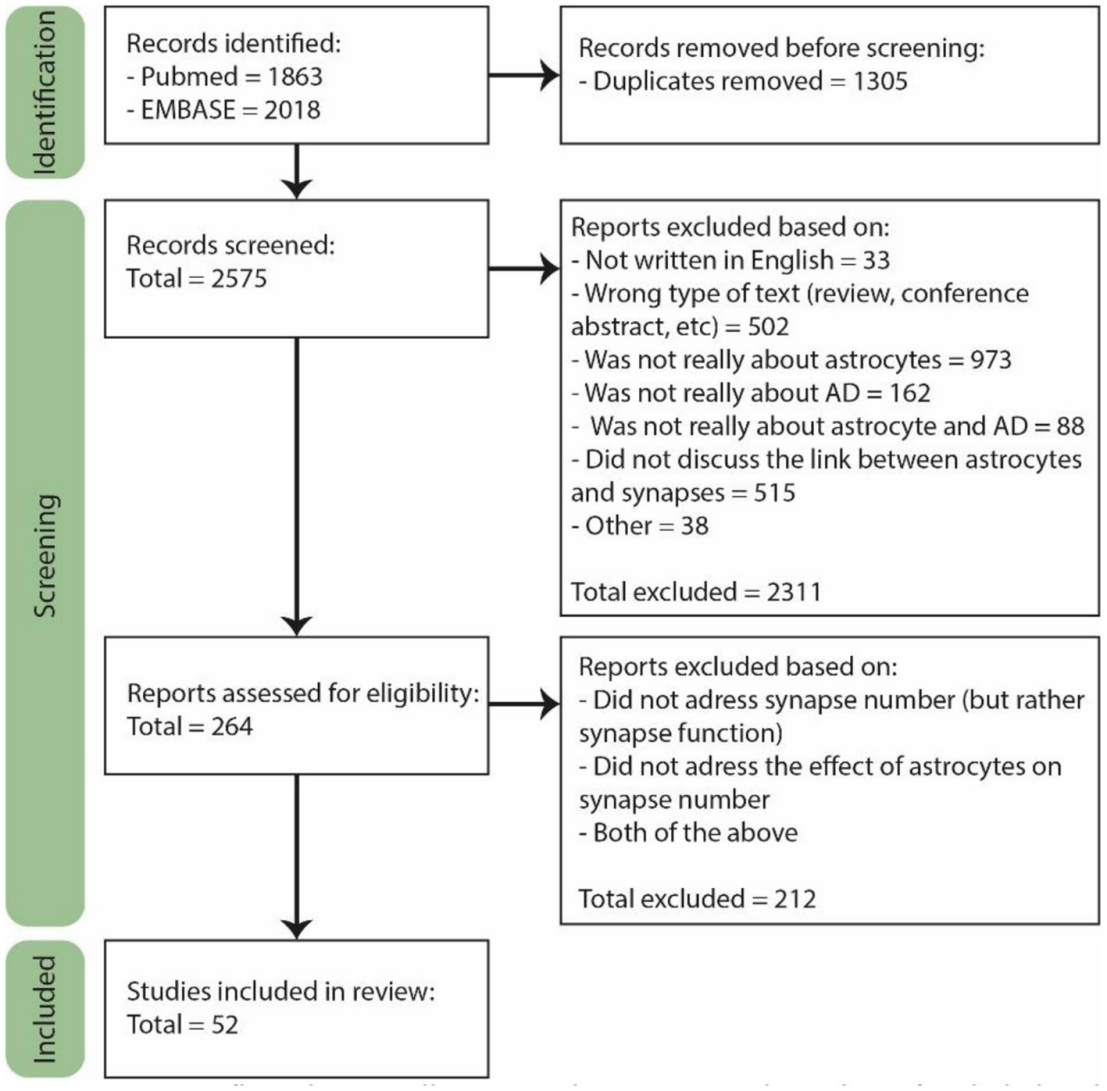
PRISM flow diagram illustrating the process and number of included and rejected papers throughout selection. During each step of the selection process papers were excluded based on several exclusion criteria. Figure adjusted from Page et al. (2021).

The last search was performed on February 2nd 2022. For the selection, we used the Rayyan online tool (Figure 1, Page et al., 2021). A total of 2,575 papers were found of which 264 were included for full text screening because their title or abstract suggested that astrocyte functioning in AD was discussed in the paper. Furthermore, only primary research articles written in English were included, though several reviews were used for general information. Both human and rodent studies were included, and although we did not actively exclude other animal models, they are not included in our final selection. From the 264 papers, the majority did not discuss the number of synapses, but rather synapse function or signaling. In the end, only the papers specifically discussing the effect of astrocytes on the number of synapses in AD or a model thereof were included, which was a total of 52 papers.

## Results

### Overview of Discussed AD Models

The systemic literature search yielded papers reporting on a variety of different AD model systems that can each give insight into how astrocytes affect synapse number in AD. In total *four* different types of AD *in vitro* models were used: (1) primary cell cultures isolated from wild-type (WT) rat or mouse brain tissue where neurons or astrocytes are treated with Aβ to induce an AD-like phenotype, (2) primary cell cultures isolated from an AD animal model, (3) control human embryonic stem-cell derived or human induced pluripotent stem cell (iPSC)-derived neuron-astrocyte co-cultures treated with ApoE protein, and (4) human iPSCs with different ApoE genotypes.

Most studies were performed using rodent models, either by injecting Aβ into brains or by using transgenic mice expressing humanized genes or genetic mutations linked to AD. Most of these models focus either on Aβ pathology, with changes in the amyloid precursor protein (APP) and/or presenilin gene, or on tau pathology with a mutation in the microtubule-associated protein tau (MAPT). Only two models that are discussed show both Aβ and tau pathology, namely the 3xTg and the 5xFAD mouse models for AD. Also, transgenic ApoE mice are used, which express different human ApoE isoforms in neurons and/or astrocytes, or ApoE knock-out mice. These ApoE transgenic mice are also crossed with the APP/PS1 or tau AD mouse models, enabling to study the interaction between different ApoE isoforms and different aspects of AD pathology.

Several studies use post-mortem tissue from AD patients to look at protein expression levels and patterns in different cell types, and link this to differences in synapse number. By including information from a neuropsychological test, such as the commonly used CERAD score, it is possible to correlate these expression patterns with cognitive performance. Only by combining information obtained with all these AD models a complete overview of whether astrocytes affect synapse number in AD can be obtained.

For the remainder of this review, astrocytes in post-mortem AD brains, astrocytes derived from AD animal models, astrocytes stimulated with Aβ and astrocytes that express ApoE4, are referred to as “AD astrocytes.”

### AD Astrocytes Reduce the Number of Synapses

During AD pathogenesis, astrocytes become reactive: their morphology becomes hypertrophic and their gene expression profile changes (for an extensive review on reactive astrocytes, see Escartin et al., 2021). Several studies showed a negative correlation between astrocyte reactivity and number of synapses in AD. For example, in the cortex and hippocampus of APP/PS1 mice the expression of GFAP was increased while expression of synaptophysin was decreased (Qiao et al., 2016). The importance of astrocytes in AD-related neuronal and synaptic loss was further shown by treating primary rat hippocampal neurons with Aβ in the presence of healthy astrocytes or Aβ-treated astrocytes. Whereas, healthy astrocytes protected against synaptic loss, Aβ-treated astrocytes decreased synaptic protein expression in neurons (Paradisi et al., 2004). Similar effects were observed when treating cultured neurons with astrocyte-conditioned medium (ACM). Treatment of primary mouse hippocampal neurons with ACM significantly reduced Aβ mediated decrease in synapse density, as measured by immunoreactivity of spinophilin, synaptophysin, or post-synaptic density 95 (PSD-95). Treatment with ACM from Aβ treated astrocytes had only a limited protective effect. Those effects were seen with both ACM derived from human and murine astrocytes (Pereira Diniz et al., 2017). Previously in our lab, acutely isolated astrocytes from the cortex of APP/PS1 mice were used for transcriptional comparison with age-matched control astrocytes (Orre et al., 2014). Astrocytes from the APP/PS1 mice showed a more proinflammatory phenotype, with reduced expression of neuronal and synaptic support genes. This data is supported by another study showing that reactive astrocytes as observed in AD had a decrease in protein expression linked to neuronal survival and synaptogenesis (Liddelow et al., 2017). A lot can also be learned from studying the brains of people who are resilient to AD. They do not show neuronal and synaptic loss despite having high levels of amyloid plaques and neurofibrillary tangles. Compared to the brains of AD-patients, their brains displayed lower levels of astrocyte activation, as was shown by a decrease in GFAP (Perez-Nievas et al., 2013). To examine the effect of glial reactivity on synapse loss, an anti-inflammatory drug called MW-151 was administered to APP/PS1 mice. MW-151 is a selective suppressor of Aβ-induced glia proinflammatory cytokine production and suppressed both astrocyte and microglia activation (Bachstetter et al., 2012). MW-151 treatment prevented loss of synapses, as measured by western blots for the levels of PSD-95, synaptophysin, syntaxin, and synaptosomal-associated protein 25 (SNAP25), without affecting amyloid plaque load or soluble Aβ concentrations. Altogether, these experiments suggest that astrocyte reactivity could be a leading factor in the loss synapses.

We have identified several pathways or processes that are altered in AD astrocytes and we will describe how this may affect synapse number (Table 1). We distinguish between direct (A) and indirect synapse loss (B). Direct synapse loss includes phagocytosis or pruning by astrocytes in AD, which we view as active processes of synapse elimination. Indirect synapse loss refers to a change in astrocyte functioning that affects the astrocyte's ability to provide the environment needed for synapse growth or changes that lead to increased synaptotoxicity. Both mechanisms will be discussed below.

**Table 1:** Astrocyte proteins involved in direct and indirect synapse elimination.

**Astrocyte protein**	**Astrocyte expression changes in AD**	**Effect on synapses in AD**	**Model**	**References**
**Direct synapse elimination**				
MERTK	Down-regulation in AD	Reduced phagocytosis of damaged synapses	12-month-old APP751_sl_ mice, on a C57BL6 background, sex unknown	(Sanchez-Mico et al., 2020)
MEGF10	Down-regulation in AD	Reduced phagocytosis of damaged synapses	12-month-old APP751_sl_ mice, on a C57BL6 background, sex unknown	(Sanchez-Mico et al., 2020)
C3	Increased expression in AD	Induced synapse loss near plaques and stimulated neurodegeneration due to tau.	6- and 9-month-old male and female PS2APP and 9-month-old TauP301S mouse models crossed with C3KO mice, background unknown, and in post-mortem AD brain tissue	(Wu et al., 2019)
	–	Enhanced Aβ mediated loss of synapses and neurons	16-month-old male APPswe/PS1dE9 and APPswe/PS1dE9;C3-KO mice, on a C57BL6 background	(Shi Q. et al., 2017)
ApoE4 (compared to effect of ApoE3)		Decreased spine density and shorter spines	Astrocyte neuron co-cultures from E17 or P2/3 ApoE4 transgenic mice on a C57BL6 background	(Nwabuisi-Heath et al., 2014)
		Increased tau-induced synapse loss	9.5-month-old female P301S Tau/Aldh1l1-Cre/apoE4^flox/flox^ mice, on a C57BL6 background	(Wang et al., 2021)
		Reduced PSD-95 and Synapsin-1 expression	Human iPSC models	(Zhao et al., 2017)
		Increase in spine density	Stem cell-derived human cell cultures	(Huang et al., 2017, 2019)
		Decreased phagocytosis of synapses, thereby increasing number of non-functional synapses	129P2-ApoE^tm3(APOE*4)*Mea*^ M8 Knock-In mice, on a C57BL6 background, age and sex unknown	(Chung et al., 2016)
		Impaired Aβ uptake and cholesterol accumulation	iPSCs sporadic AD human, with ApoE3 and 4 genotypes, differentiated into neurons and astrocytes	(Lin et al., 2018)
		Increased tau mediated synapse loss	9-month-old male P301S/ApoE3 or ApoE4 Knock-In, or ApoE KO mice, on a C57BL6 background	(Shi Y. et al., 2017)
**In-direct synapse elimination**				
GLT1	Reduced expression in AD	–	Post-mortem human brain tissue	(Jacob et al., 2007; Scott et al., 2011; Kobayashi et al., 2018)
	Reduced expression in AD	–	6-month-old APP/Ld/2 mice, on a C57BL6/FVB/N background, sex unknown	(Masliah et al., 2000)
	Expression decreased by Aβ	Possibly resulting in excitotoxicity*	Primary cortical astrocyte cultures from 4 to 5-postnatal-days old Wistar rats, treated with Aβ	(Matos et al., 2008)
			3-month-old male Swiss mice, injected with Aβ in the brain	(Mahmoud et al., 2019)
	Pharmacologically Increased GLT1 expression	Increased synaptophysin and PSD-95	12–14-month-old APP/EAAT2 mice, on a C57BL6 background, sex unknown	(Wu et al., 2019)
			12-month-old male and female 3xTG mice, on a C57BL6/129SvJ background	(Zumkehr et al., 2015)
			5–6-months-old male and female APP/PS1 mice, on a C57Bl/6 background	(Hefendehl et al., 2016)
GLAST	Reduced expression in AD	Possibly resulting in excitotoxicity*	6-month-old APP/Ld/2 mice, on a C57BL6/FVB/N background, sex unknown	(Masliah et al., 2000)
Glutamine synthetase	Decreased expression in AD	Possibly resulting in excitotoxicity*	1–9-month-old male 3xTg-AD mice, on a C57BL6 background	(Kulijewicz-Nawrot et al., 2013)
A2AR	Elevated in AD brains	–	14–20-month-old APP and 16.5-month-old APP/PS1 mice, on a C57BL/6J background, and post-mortem human brain tissue	(Orr et al., 2015)
		Decreased expression of GLT1 and GLAST, thereby decreasing glutamate uptake, possibly resulting in excitotoxicity*	Primary mouse cortex cell cultures, from day 4–5 postnatal C57BL/6-A_2A_R-GKO mice.	(Matos et al., 2012)
			2–3-month-old male Gfa2-A2AR-KO mice, on a C57BL/6 background	(Matos et al., 2013)
GluN2A		Reduced loss of PSD-95 and Synaptophysin	Primary rat hippocampal cell cultures from postnatal day 4 Wistar rats	(Li et al., 2016)
			Aβ injections in DG of male Wistar rats, with GluN2A knockdown, age unknown	(Du Z. et al., 2021)
TSP1	Expression is decreased in AD	Loss of synaptophysin and PSD-95	Primary rat cortical cultures from 1 to 2-day-old rats, strain and sex unknown	(Rao et al., 2013)
	Artificially increased expression	Rescued Aβ induced spine loss	12-month-old Tg2576 mice, on a B6SJL background, sex unknown	(Son et al., 2015)

^*^The excitotoxicity is our hypothesis and is not shown in the corresponding literature.

### Direct Synapse Elimination Through Synaptic Pruning

#### Phagocytic Capacity of AD Astrocytes

Both *in vitro* (Chung et al., 2013) and *in vivo* (Lee et al., 2021) studies with WT adult mouse models have shown that astrocytes play an important role in the phagocytosis of synapses throughout the brain. Dysregulation of phagocytosis has been associated with AD, although most studies focused on the involvement of microglia (Galloway et al., 2019; Nizami et al., 2019) and overlooked the involvement of astrocytes. In and surrounding Aβ plaques astrocytic processes, microglia processes, Aβ fibers, and dystrophic neurites are all very tightly packed together. Electron microscope imaging of APP/PS1 mouse model brain slices, showed that the reactive astrocytes enwrap the dystrophic neurites and engulf them (Gomez-Arboledas et al., 2017), thereby phagocytizing and degrading presynaptic structures. There was an age-related increase in the number of dystrophic presynaptic structures engulfed by astrocytes, as well as an increase in engulfed dystrophies per plaque. However, the proportion of dystrophic presynaptic structures engulfed by astrocytes in respect to the total number around plaques was relatively low, and with disease progression the presence of dystrophies continues to accumulate. This suggests that the presence of Aβ plaques limits or impairs the phagocytic capacity of the reactive astrocytes and microglia. This hypothesis is strengthened by the observation that primary astrocyte cultures obtained from 12-month-old APP751α mice had a highly reduced phagocytic and degradative capacity of isolated synapses, as compared to primary astrocyte cultures from WT or Tau P301S mice (Sanchez-Mico et al., 2020). Adding Aβ oligomers reduced the phagocytic and degradation capacity of primary astrocytes from WT mice, indicating that Aβ directly impairs the capacity of astrocytes to clear the pathological markers of AD. Also *in vivo*, in the hippocampus of 12 months old APP mice, only a very small number of reactive astrocytes engulfed dystrophic synapses (Sanchez-Mico et al., 2020).

The two main secreted proteins by astrocytes that are involved in phagocytosis of synapses are multiple EGF-like domains 10 (MEGF10) and c-mer proto-oncogene tyrosine kinase (MERTK). Both *in vitro* and *in vivo* phagocytosis of synapses by astrocytes was measured by super-resolution imaging and scanning electron microscopy to visualize engulfment of synaptic material. Knock-out of either MERTK or MEGF10 in astrocytes resulted in a 50% reduction of their phagocytic capacity of synaptosomes, and knocking-out both genes even further reduced phagocytosis, showing that MERTK and MEGF10 work in parallel to mediate synapse elimination (Chung et al., 2013). Interestingly, there was a decrease in MERTK and MEGF10 expression in the hippocampus of 12 months old APP751α mice as compared to the expression at 3 months (Sanchez-Mico et al., 2020). Other studies showed only minor changes in MERTK and MEGF10 expression with normal aging (Chung et al., 2013; Boisvert et al., 2018), which suggests an AD-related decrease in MERTK and MEGF10 expression in the APP751α mice. It could be that progression of AD results in a decrease in MERTK and MEGF10 expression, which in turn results in reduced phagocytosis of damaged synapses, leading to an increase in dystrophic neurites and possibly a more synaptotoxic environment.

ApoE isoforms differentially affect astrocytic phagocytosis: *in vivo* experiments with transgenic mice expressing different ApoE isoforms showed that compared to the ApoE3 isoform, ApoE4 expressing astrocytes have a slower phagocytosis rate, which leads to more non-functional synapses (Chung et al., 2016). This change in phagocytosis was inversely correlated with C1q expression in the hippocampus, as in the ApoE4 mice the C1q expression was increased compared to ApoE3 (Chung et al., 2016). C1q is a marker for complement activity and microglia phagocytosis but is also associated with senescent synapses, and analyzing immunofluorescent colocalization of synaptophysin and PSD-95 showed that blocking C1q activity could prevent synapse loss in early stages of AD (Hong et al., 2016). This suggests that ApoE4 expression leads to decreased elimination of senescent synapses.

Overall, several lines of evidence indicate that AD astrocytes show deficits in the elimination of senescent synapses. Although this initially leads to a potential increase in synapses, these synapses are predominantly malfunctioning, and over time, the accumulation of senescent synapses could result in an unhealthy synaptic environment, causing deficits and memory loss.

#### Increased Complement Expression in AD, Linked to Increased Synapse Loss

Microglia are most commonly viewed as the key players in phagocytosis in the brain. However, astrocytes can influence microglia-mediated synaptic pruning through activation of the complement cascade. For instance, AD-associated decrease in astrocyte expression of glutamate transporter 1 (GLT1/EAAT2) increases C1q production and enhances microglial phagocytosis of synapses in WT rats injected with Aβ (Wu et al., 2020). As part of the immune system, the complement system helps clear pathogens, regulates the inflammation response and is involved in synaptic pruning (Stevens et al., 2007). In CSF and blood samples of AD patients, the activity of the complement system was increased (Krance et al., 2019). C3 plays a central role in activation of the complement system. In both the PS2APP and Tau P301S AD mouse models, astrocytic complement C3 protein and mRNA expression was increased throughout the brain, specifically in the hippocampus (Wu et al., 2019). Microglial and neuronal C3 mRNA expression was low compared to astrocytic expression, and only in the Tau P301S mouse model changes in microglia C3 expression were observed compared to WT controls. Also in human AD brains C3 levels were increased, both near synapses and in the CSF (Wu et al., 2019). When complement activity was blocked by crossing either the PS2APP or the Tau P301S AD mouse model with the constitutive C3 knock out (KO) mouse model, reduced loss of PSD-95 staining near plaques in the PS2APP mice and limited neurodegeneration in the Tau P301S mouse model was observed. This suggests that complement activity, most likely astrocytic C3 expression, increases the elimination of synapses and neurons in AD.

Another study showed that inhibition of the complement pathway by blocking C1q, C3, or the microglial complement receptor CR3, reduces microglia phagocytosis and early synapse loss (Hong et al., 2016). KO of C3 in an APP/PS1 mouse model protected against Aβ mediated loss of neurons (Shi Q. et al., 2017). It is important to note that both microglia and astrocytes express C3, and after KO of C3, both cell types show a reduced co-localization with Aβ. Therefore, based on these experiments it cannot be deduced whether the observed effects are primarily due to changes in astrocyte or microglia activity, or their interaction. Since this review focuses on the direct interaction between astrocytes and synapses, we will not discuss the role of microglia in synapse loss in much detail. For more information on microglia-synapse interaction see Schafer et al., 2013.

### Reduced Support and Formation of Synapses, and Increased Synaptotoxicity

#### Neurotoxicity Due to Impaired Glutamate Transport and Signaling

Astrocyte processes closely enwrap synapses and play an important role in the regulation of the extracellular environment in the synaptic cleft. Essential for this regulation is the recycling of neurotransmitters. Glutamate is the main excitatory neurotransmitter. It is constantly produced and released into, and then recycled from the synaptic cleft. Astrocytes are key regulators of glutamate homeostasis, by balancing between glutamate uptake and release (Mahmoud et al., 2019). Proper transport and recycling of glutamate are of utmost importance, as excessive glutamate in the synaptic cleft could lead to excitotoxicity and synapse loss. Several transporters regulate the reuptake of glutamate from the synaptic cleft. Changes in glutamate transport and recycling have been implicated in AD; most studies show a decreased expression of glutamate transporters or glutamine synthetase in the brains of AD patients (Masliah et al., 2000; Scott et al., 2011) and AD animal models (Minkeviciene et al., 2008; Kulijewicz-Nawrot et al., 2013). Here we will discuss how astrocytes might play a role in the altered regulation of glutamate in AD.

#### GLT1 and GLAST

Two important glutamate transporters that are mainly expressed by astrocytes are GLT1, and glutamate-aspartate transporter (GLAST/EAAT1). Post-mortem brain tissue with AD pathology, both Aβ plaque and tau tangles, was compared between individuals with and without cognitive dysfunction. It was shown that in the brains of donors with cognitive dysfunction the GLT1 expression was decreased, while in brains of donors without cognitive dysfunction GLT1 expression was similar to donors without AD pathology (Jacob et al., 2007; Scott et al., 2011; Kobayashi et al., 2018).

*In vitro* it was shown that Aβ decreases glutamate uptake by primary cortical rat astrocytes by reducing GLT1 and GLAST activity (Matos et al., 2008). In addition, other studies showed an increase in astrocyte glutamate release after exposure to Aβ (Talantova et al., 2013). So, Aβ both inhibits the reuptake of glutamate by astrocytes, and increases the release of glutamate from astrocytes. This could lead to excessive glutamate in the synaptic cleft, which in turn could lead to synaptic loss. This hypothesis is supported by research using different mouse models for AD (APP/PS1, 3xTg and APPsw/Ind), where it was shown that when GLT1 expression is pharmacologically increased, levels of synaptophysin and PSD-95 increase (Zumkehr et al., 2015; Hefendehl et al., 2016), and even cognitive function improves (Takahashi et al., 2015).

Glutamate recycling by GLT1 and GLAST is also indirectly affected *via* altered adenosine A2A receptor (A2AR) expression. A2ARs are expressed by astrocytes and enhance the release of several neurotransmitters. Activation of A2AR using an agonist decreased expression of GLT1 and GLAST and thereby decreased glutamate uptake (Matos et al., 2012, 2013). A2AR expression is elevated in the brains of AD patients and of an AD mouse model, and when A2AR was conditionally knocked out in AD mice, those mice showed enhanced memory performance (Orr et al., 2015).

#### NMDARs

N-methyl-D-aspartate receptors (NMDARs) also play a role in glutamate signaling. Although they are mainly expressed by neurons, astrocytes also express NMDARs (Conti et al., 1996). Using DiI labeling and confocal imaging, it has been shown that by blocking two major NMDAR subunits (GluN2A and GluN2B) on astrocytes, Aβ induced synapse loss was aggravated in co-cultures of astrocytes and neurons (Li et al., 2016), and activation of astrocyte NMDARs reduced loss of PSD-95 and synaptophysin. These results were validated *in vivo*. Astrocyte-specific knock-down of GluN2A was combined with Aβ injection in the DG of rats, and showed an aggravation of Aβ-induced decrease of PSD-95 and synaptophysin immunoreactivity, along with increased impairment of spatial memory and cognition (Du Z. et al., 2021). Experiments with NMDA antagonists in mice treated with Aβ injection showed a decrease in synaptophysin immunostaining and cognitive impairments (Bicca et al., 2011). Contradictory results were found when treating yellow-fluorescent-protein (YFP) expressing 3xTG mice with the NMDAR antagonist NitroMemantine. These mice showed a significant increase in synaptic and dendritic density (Talantova et al., 2013). However, NitroMemantine is designed to block neuronal NMDAR, suggesting a possible opposing role for astrocytic and neuronal NMDAR in Aβ mediated excitotoxicity. Only a limited number of studies examines NMDARs in astrocytes, and it is unknown whether astrocytic NMDAR expression changes in AD. NMDAR activity in AD could also be affected in a different way. Reactive astrocytes express serine racemase (SR), an enzyme that converts L-serine to D-serine (Li et al., 2018). Both post-mortem AD brain tissue and the TgF344-AD rat model showed high expression levels of SR (Balu et al., 2019). D-serine is a co-agonist for NMDARs, and it was shown that D-serine release from reactive astrocytes induced excitotoxicity and decreases neuronal survival. Future studies are needed to look into this and clarify the possible important role of astrocytic NMDAR in AD.

Overall, multiple lines of evidence indicate that normal astrocyte functioning is crucial for glutamate regulation and protects against Aβ induced synapse loss and that those processes are affected in AD. As a consequence, glutamate transport is hindered, which could be the cause of synaptotoxicity. Impaired glutamate transport could also affect activation of NMDARs, which could also lead to aberrant synaptic function and result in synapse loss.

#### Impaired Stimulation of Synapse Formation by Thrombospondins

Thrombospondins (TSPs) are extracellular multidomain glycoproteins that affect cell-matrix interactions (Adams, 2001). TSPs are a gene family of five members: TSP1–TSP5, each of which has a distinct expression pattern over different tissues and throughout development. Astrocytes secrete two TSPs, TSP1 and TSP2, which can induce synapse formation. Mice without TSP1 and TSP2 had a 25–30% reduction in the number of excitatory synapses in the cortex (Christopherson et al., 2005). Furthermore, *in vitro*, the addition of TSP1 or TSP2 to rat retinal ganglion cells cultured in serum-free conditions significantly increased the number of spines in a dose-dependent manner.

In post-mortem brain tissue of AD patients and in the hippocampus of both the Tg2576 and the Tg6799 AD mouse model, a reduced TSP1 expression was found, whereas the TSP2 expression was not changed (Son et al., 2015). Furthermore, treatment of primary rat cortical astrocytes with Aβ caused a decrease in TSP1 release from the astrocytes and a correlated increase in intracellular TSP1. Culturing primary rat cortical neurons with TSP1 depleted ACM resulted in a reduction of synaptophysin and PSD-95, as compared to non-treated neurons, while treatment with normal ACM increased PSD-95 expression (Rao et al., 2013). Both *in vitro* and *in vivo* it was shown that adding TSP1 protein attenuates Aβ mediated synapse loss. Cotreatment of Aβ with recombinant TSP1 protein preserved PSD-95 levels and the number of spines, and injection of TSP1 protein in Tg2576 and Tg6799 AD mice increased expression of several synaptic proteins, such as PSD-95 and NR2A (Son et al., 2015).

In conclusion, Aβ reduces the secretion of TSP1 by astrocytes in the AD brain. As a consequence, there is decreased synapse formation, which leads to a decrease in synapse number.

#### The Effect of ApoE Isoform Expression in Astrocytes on Synapse Number

Several *in vitro* and *in vivo* experiments have been performed that look into the effect of ApoE4 expression on synapse number compared to ApoE3 expression. When co-culturing astrocytes from homozygous E3 and E4 transgenic mice with wild-type neurons, the presence of ApoE4 astrocytes decreased spine density compared to co-cultures with E3 astrocytes (Nwabuisi-Heath et al., 2014). Similarly, neurons co-cultured with ApoE4 expressing human iPSC-derived astrocytes showed a reduction in survival rate and less PSD-95 expression compared to neurons co-cultured with ApoE3 expressing human iPSCs-derived astrocytes (Zhao et al., 2017). The opposite effect was found in stem cell-derived human neurons and glia (not further specified by the authors, but most likely from an astrocyte origin) cell cultures; adding different glia-derived ApoE variants to neuron cultures grown without glia significantly increased synapse density compared to neuron cultures without ApoE (Huang et al., 2019). Here, the addition of ApoE4 showed the biggest increase in synapses, and the addition of ApoE2 the smallest. These results were found using confocal imaging of synapsin and Homer staining, and further validated with western blot analysis of synapsin, PSD-95, synaptophysin, synaptotagmin, and synaptobrevin. Yet another experiment with iPSCs found an increase of the immunofluorescent signal of synaptophysin and PSD-95 in ApoE4 expressing neurons, while astrocytic ApoE4 expression was linked to impairments in Aβ clearance and lipid transport, but not to changes in synapse number (Lin et al., 2018). Most *in vivo* models seem to suggest that ApoE4 expression negatively affects synapse number, however, whether this is due to neuronal or astrocytic ApoE expression is unclear. Conditional KO of astrocytic ApoE4 in a tau mouse model for AD decreased tau pathology and tau-induced brain atrophy and synaptic loss (Wang et al., 2021). These experiments suggest that astrocyte ApoE4 expression enhances synapses loss. Other experiments, using Golgi stainings to analyze synapse density and morphology in transgenic ApoE mice showed that astrocytic expression of different ApoE isoforms did not affect spine density or morphology, while neuronal knockin (KI) of ApoE4 resulted in a loss of dendritic spines, specifically in mushroom-shaped spines (Jain et al., 2013). Besides astrocytes and neurons, also microglia express ApoE (Xu et al., 2006). Taken together, it is not yet completely clear what effect the ApoE4 isoform from different cellular sources has on synapse number, and more studies are needed to clarify this.

The molecular mechanisms as to how different ApoE isoforms affect AD are not completely understood. ApoE4 increases the pro-inflammatory response, which could result in dysfunction of the blood brain barrier (BBB) (Teng et al., 2017). Different ApoE isoforms also differently affect lipid binding, which in turn affects cholesterol concentrations and glucose utilization (reviewed in Yassine and Finch, 2020). In addition, ApoE4 also directly affected neuropathological markers of AD compared to ApoE3: it enhanced tau protein aggregation and impaired Aβ uptake and aggregation (Huang et al., 2017; Shi Y. et al., 2017; Lin et al., 2018; Chen et al., 2021). Glial ApoE4 increased Aβ production compared to ApoE3, and neurons cultured without ApoE secreted from glia secrete 2- to 3-times less Aβ. Increased Aβ production could result in synapse loss. ApoE binds to Aβ, the strength of which is dependent on ApoE-isoform (Strittmatter et al., 1993). This in turn affects Aβ aggregation: ApoE4 derived from neurons slows aggregation the most compared to ApoE3 and ApoE2. However, ApoE isoforms derived from astrocytes did not show an isoform-specific difference in how they affect Aβ aggregation. For a more extensive review on this topic, see Chen et al., 2021.

Altogether, ApoE4 seems to affect cellular processes in such a way that results in an unhealthy environment, which could be harmful for synapses, thereby indirectly causing synaptic loss. Whether these effects are due to astrocytic, neuronal, or microglial ApoE remains to be determined.

### Other Astrocyte Proteins That Affect Synapse Formation and Elimination

From our systematic search, several studies were found that reported on astrocytic proteins or secreted molecules that may affect synapse formation and elimination, but their roles in AD remain speculative. These studies are summarized in Table 2. We distinguish three categories, those which report on (i) an increased expression in AD, (ii) a decreased expression in AD, and (iii) with no information on the expression in AD, but changed levels affect synapses.

**Table 2:** Increased and downregulated astrocyte proteins that affect synapse number.

**Astrocyte protein**	**Astrocyte expression changes in AD**	**Effect on synapses in AD**	**Model**	**References**
**Increased expression in AD**				
ATP	Release increased by Aβ	Protects against Aβ mediated spine loss	Primary mouse astrocytes from 1-day-old ICR mice, human U373 astrocyte, and primary rat hippocampal cell cultures, strain unknown, treated with Aβ	(Jung et al., 2012)
LCN2	Brain-region specific upregulation	–	Post-mortem AD patient brain tissue	(Dekens et al., 2017)
	Increased expression after Aβ treatment;	–	Primary cortex cultures of astrocytes from 5 to 7-day-old Wistar Han rats, treated with Aβ, sex unknown	(Mesquita et al., 2014)
	Increased expression after Aβ treatment;	Reduced total spine number, specifically affecting thin and mushroom spines	Primary cortex of hippocampal astrocyte culture from 0 to 3-day-old C57BL/6 pups and organotypic hippocampal slice cultures from 6 to 8-day-old mice, treated with Aβ	(Maysinger et al., 2017)
CaN	Increased expression in reactive astrocytes.	–	3–18-month-old male APP/PS1 mice, on a C57CBL/6J background	(Norris, 2005)
	–	Inhibition increases levels of synaptophysin and PSD-95	10-month-old male and female AβPP/PS1 mice, on a B6C3 background	(Hong et al., 2010)
P2Y1R	Increased in AD patients and AD mouse model.	Blocking P2Y2R activity attenuates AD induced loss of synapses	Post-mortem brain tissue and aged (8+-month-old for expression, and 11-month-old for synapse analysis) APP/PS1 mice, on a C57BL/6J background, sex unknown	(Reichenbach et al., 2018)
CIP2A	Upregulated expression in reactive astrocytes (1)	CIP2A induced astrocyte reactivity decreased PSD-95, synapsin-1 (2) and synaptophysin levels and CIP2A overexpression in astrocytes resulted in decreased number of dendritic spines in hippocampus (3)	(1) Post-mortem AD brain tissue and 9-month-old male 3xTg mice, unknown background (2) Primary rat cortical astrocyte and neuron culture from new-born Sprague-Dawley rats (3) 9-month-old male 3xTg mice, unknown background	(Shentu et al., 2019)
TRPA1	Increased expression in hippocampus	–	8-month-old APP/PS1 mice, on a C57BL background, sex unknown	(Lee et al., 2016)
	–	Inhibition of TRPA1 prevents astrocytic withdrawal from spines and preserves spine density and morphology.	1–3-month-old male and female APP/PS1-21 mice, on a C57BL6/J background	(Paumier et al., 2021)
**Decreased expression in AD**				
BDNF	Decreased levels in AD	Decreased spine density and PSD-95 and synaptophysin expression	Hippocampus of 5-monht-old Tg2576, on a B6SJL background, sex unknown	(Hongpaisan et al., 2011)
			Primary hippocampal astrocyte-neuron co-cultures from 1 to 3-day-old BDNF KO mice 8-month-old 5xFAD male mice crossed with BDNF KO mice or pGFAP-BDNF mice, background unknown	(Du Z. et al., 2021)
TGF-β1	Decreased expression by Aβ	Increased Aβ mediated spine loss. Treatment with TGF-β1 prevents Aβ induced synapse loss.	Primary mouse hippocampal cultures from 1 to 2-day-old Swiss mice, adult primary human astrocyte cultures and 3-month-old male Swiss mice	(Pereira Diniz et al., 2017)
**Unknown expression change in AD**				
5-HT_2_ receptor	?*	When active prevents loss of Synaptophysin and MAP2 *via* reduction of astrocyte Aβ production	Hippocampal neuron cultures treated with ACM from FLX treated primary cortical astrocyte cultures from 1-day-old APP/PS1 mice, sex unknown	(Qiao et al., 2016)
FOXO3	?*	FOXO3 KO results in a loss of synaptophysin and PSD-95	Cortex of 3.5-month-old male and female 5xFAD mice, on a C57BL/6J background	(Du S. et al., 2021)

^*^Unknown expression change in AD.

When cultures of primary astrocytes and U373 human glioblastoma cells were treated with Aβ, the release of adenosine triphosphate (ATP) was increased. Spine density analysis of phalloidin-stained rat primary hippocampal neurons showed that pre-treatment with ATP protected against Aβ mediated synapse loss *via* interaction with P2 purinergic receptors (Jung et al., 2012). This is in contrast with most proteins that are differentially expressed or secreted by AD astrocytes, which have a negative effect on synapse number. For example two studies showed that Aβ treatment increased expression of lipocalin-2 (LCN2) by astrocytes (Mesquita et al., 2014; Maysinger et al., 2017). This reactive astrocyte secreted protein was also upregulated in specific brain regions of AD patients, such as the hippocampus, amygdala and several, but not all cortical regions (Dekens et al., 2017). Imaging of mouse hippocampal slice cultures from transgenic mice that express green fluorescent protein (GFP) under an axonal promotor showed that addition of recombinant LCN2 reduced the total number of spines, and treatment with anti-LCN2 rescued Aβ mediated spine loss, suggesting that increased LCN2 expression by AD astrocytes negatively affects synapse number (Maysinger et al., 2017). Similarly, cancerous inhibitor of PP2A (CIP2A) is highly expressed in astrocytes, and upregulated in AD patients, as well as in the 3xTg mouse model (Shentu et al., 2019). Virus-induced overexpression of CIP2A by astrocytes *in vivo* reduced the number of dendritic spines and decreased the expression levels of synaptic genes in the hippocampus, but not in the cortex, as shown by both reduced protein expression of PSD-95, synapsin, and synaptophysin analyzed with western blot experiments, and synapse density count based on a Golgi staining.

Astrocytic Ca^2+^ signaling is emerging as a key component of signal processing in the brain and aberrant astrocytic Ca^2+^ signaling in AD is likely to affect synapse function (Guerra-Gomes et al., 2018). The metabotropic receptor P2Y1R increases calcium events in astrocytes upon interaction with ATP and ADP (Delekate et al., 2014). Its expression is increased in reactive astrocytes in AD patients and 8 month and older APP/PS1 mice, and colocalization analysis of Homer and synaptophysin showed that chronic treatment with a P2Y1R antagonist prevented the decrease in synaptic density that is normally observed in these mice (Reichenbach et al., 2018). Calcineurin (CaN), a calcium-sensitive serine/threonine phosphatase, is expressed in neurons, but its expression is increased in reactive astrocytes, for example in 3 to 18 month old APP/PS1 mice (Norris, 2005). Inhibiting CaN activity in these mice not only reduced Aβ plaque burden and astrocyte reactivity, but western blot analysis also showed increased expression of synaptophysin and PSD-95 (Hong et al., 2010), suggesting a role for increased CaN in AD astrocytes in synapse loss. In line with this, the expression of transient receptor potential ankyrin 1 (TRPA1), an astrocyte calcium channel, is increased in the hippocampus of 8 months old APP/PS1 mice (Lee et al., 2016). Chronic inhibition of TRPA1 in APP/PS1-21 mice that express YFP under control of a axonal promotor showed preserved spine density and morphology (Paumier et al., 2021). Overall, these studies show that normalizing astrocytic calcium signaling could prevent synapse loss.

Although many proteins are increased in AD astrocytes, some are decreased which also leads to reduced spine density. For instance, astrocyte secreted transforming growth factor β1 (TGF-β1), known to stimulate synapse formation, was decreased by the presence of Aβ (Pereira Diniz et al., 2017). Western blot analysis of PSD-95 and synaptophysin expression showed that adding TGF-β1 protected against Aβ induced spine loss, and pre-treating mice with TGF-β1 before Aβ injection fully prevented the decrease of synaptic proteins. Also, levels of brain-derived neurotrophic factor (BDNF) were decreased in Tg2576 (Hongpaisan et al., 2011) and 5xFAD mice (de Pins et al., 2019). Although this decrease was not shown specifically for astrocytes, primary mouse hippocampal neurons co-cultured with BDNF-deprived astrocytes had a decreased spine density, and engineered astrocytes that deliver BDNF under the control of the GFAP promotor restored dendritic spine density and morphology in 8-month-old 5xFAD mice (de Pins et al., 2019).

For other studies it is not clear whether altering expression levels in astrocytes affect synapses directly, or whether other processes are involved, such as Aβ clearance. Although neurons are seen as the major source of Aβ, astrocytes have been shown to produce Aβ (Zhao, 2011), and are implicated in Aβ degradation and clearance (reviewed in Ries and Sastre, 2016). This could be another way by which AD astrocytes indirectly affect Aβ-induced synapse loss. Fluoxetine (FLX) is a selective serotonin reuptake inhibitor used as antidepressant for AD patients. Treatment of APP/PS1 astrocyte cultures with FLX decreased Aβ levels, and adding ACM of FLEX treated APP/PS1 astrocytes to hippocampal neurons preserved synaptophysin protein levels (Qiao et al., 2016). FLX was shown to operate *via* activation of astrocyte serotonin 5-HT_2_ receptors, suggesting that astrocyte 5-HT_2_ receptor levels are important mediators in astrocyte Aβ production and subsequent synapse loss. Although studies showed a reduction of serotonin 5-HT_2_ receptors in the AD brain (Blin et al., 1993; Lorke et al., 2006), this decrease was not shown specifically for astrocytes. Forkhead box O transcription factor 3 (FOXO3) is another protein that might affect synapse number *via* astrocyte regulation of Aβ levels. 5xFAD mice with a KO of FOXO3 show a significant reduction in the number of PSD-95 and synaptophysin puncta as well as their colocalization (Du S. et al., 2021). When treating primary astrocyte cultures from FOXO3 KO mice with Aβ, there was a significant decrease in synaptic puncta. In addition, there was significantly less intracellular Aβ, suggesting a role for FOXO3 in Aβ internalization in astrocytes (Du S. et al., 2021). Viral expression of FOXO3 in the FOXO3 KO cultures restored Aβ internalization and increased both the number and colocalization of synaptophysin and PSD-95 puncta. FOXO3 is upregulated with age in the mouse cortex, however not much is known regarding changes in astrocyte FOXO3 expression in AD.

In general, AD astrocytes undergo many changes in expression and secretion of molecules, which contribute to synapse loss. Further research is needed to fully explore the effect of expression of these proteins in astrocytes on synapse formation, and how this is altered in AD.

## Discussion

Astrocytes play a crucial role in synapse formation and elimination in a healthy brain. In AD a pathological increase in synapse loss is likely to underly cognitive decline. Therefore, our systematic search aimed to show how astrocytes contribute to synapse loss in AD. We performed a systematic literature search which yielded 52 papers that discussed how AD astrocytes affect synapse number. In these papers both human and rodent studies were included, as well as *in vitro* and *in vivo* AD models.

To summarize our findings: AD induces changes in astrocytes, resulting in a reactive phenotype with changed morphology and gene expression. This AD astrocyte gene expression profile showed a more pro-inflammatory phenotype with a reduced expression of synaptic support genes. Whereas, healthy astrocytes protect against synapse loss, AD astrocytes decrease synaptic protein expression. We investigated pathways that could explain the role of AD astrocytes in AD-related synapse loss and distinguished direct synapse loss, due to an effect on phagocytosis, and indirect synapse loss due to decreased support and formation of synapses.

Astrocytes can actively affect synapse elimination through phagocytosis (Chung et al., 2013), a process most active during early development, but which continues into adulthood. Experiments have shown that the phagocytic capacity of the reactive astrocytes in AD is limited or impaired (Gomez-Arboledas et al., 2017; Sanchez-Mico et al., 2020). This could be caused by a decrease of MERTK or MEGF10 expression in AD astrocytes, but could also be *via* influencing microglia-mediated synaptic pruning. ApoE4 expressing astrocytes have a slowed phagocytosis rate, which leads to more non-functional synapses. Accumulation of senescent synapses could result in an unhealthy synaptic environment, causing deficits and memory loss. On the other hand, expression of complement activator C3 was increased in AD astrocytes (Wu et al., 2019), which is linked to increased synapse elimination. We therefore cannot conclude whether AD astrocytes increase or decrease phagocytosis of synapses, but the delicate balance between synapse formation and elimination is likely disturbed.

Synapse loss can also be caused by a decreased support for synapse formation or maintenance, or by a disruption in normal astrocyte processes leading to a synaptotoxic environment. In AD astrocytes glutamate reuptake is reduced (Matos et al., 2008) while glutamate release is increased (Talantova et al., 2013), resulting in excessive glutamate in the synaptic cleft, which could be the cause of synaptotoxicity leading to synapse loss. In addition, TSP1 expression is attenuated in AD astrocytes, which leads to less synapse formation and a decrease in synapse number (Rao et al., 2013; Son et al., 2015). Although the effects of ApoE4 expression on synapse number compared to ApoE3 expression are still under debate, most studies suggest that astrocytic ApoE4 expression affects cellular processes in such a way that results in an unhealthy environment (Nwabuisi-Heath et al., 2014; Huang et al., 2017; Shi Y. et al., 2017; Teng et al., 2017; Zhao et al., 2017; Lin et al., 2018; Wang et al., 2021), which could be harmful to synapses, thereby indirectly causing synapse loss.

As mentioned in the section “Other Astrocyte Proteins That Affect Synapse Formation and Elimination” and summarized in Table 2, many studies report AD-induced changes in gene expression that may affect synapse number, but findings are often only supported by a single study. Overall, we found that most changes in astrocyte gene expression negatively affect synapse number. AD likely initiates changes in Ca^2+^ signaling and alters Aβ production or processing in astrocytes, which could lead to a synaptotoxic environment. Follow-up studies are needed to replicate and validate these findings. In addition, as studies looking into the role of astrocytes in AD are relatively new, there are likely more astrocytic genes and pathways that affect synapse number that we will learn about in the coming years.

Many different types of models were discussed throughout this review. It is important to keep in mind that different models have different strengths and limitations. *In vitro* studies can provide information on how specific cellular processes or expressed proteins affect synapse number in an isolated system. However, usually, only a limited spectrum of the AD pathology is addressed. *In vivo* studies are better suited to study how astrocytes affect synapses in a more complex and realistic environment. Throughout this review we have addressed many papers that have analyzed the number of synapses in AD models, using variety of methods. A commonly used method is to analyze synaptic protein expression using western blot (Hong et al., 2010; Qiao et al., 2016; Shentu et al., 2019). Other papers analyzed synapse number by performing immunohistochemistry, and subsequently measuring the density or colocalization of synaptic puncta (Li et al., 2016; Lin et al., 2018; Reichenbach et al., 2018; Huang et al., 2019). Yet another method is to transfect neurons to induce expression of a fluorescent protein (Talantova et al., 2013; Pereira Diniz et al., 2017; Paumier et al., 2021), stain neurons with a dye (Li et al., 2016), or use Golgi staining (Jain et al., 2013; Shentu et al., 2019) in order to visualize the complete neuronal morphology, which can then be imaged using various microscopes. Electron microscopy is also used to identify and analyze spine density, without the aid of fluorescent staining (Hong et al., 2016). Although some papers use several of these methods and find similar results (Jung et al., 2012; Pereira Diniz et al., 2017; Shentu et al., 2019), the different methods used can also be a reason variation in outcome. Western blot analysis is on average less suited to detect subtle changes in protein expression, or in this case synapse density, while it does allow for inclusion of many cells from different brain regions. Staining, imaging and analyzing synapse density with high resolution microscopy gives a very accurate count, but is restricted to a limited number of cells, or only short sections of dendrites, that can be analyzed. Another important aspect to keep in mind is the different synaptic markers that are being used. PSD-95, synaptophysin, Homer, synapsin, syntaxin, SNAP25, spinophilin, synaptotagmin, and synaptobrevin are all synapse markers whose expression level were used to measure synapse number. Although some studies that analyze multiple markers show no differences in observed effect between those markers (Huang et al., 2019; Shentu et al., 2019), another study showed a decrease in spine number visible when measuring PSD-95 and synaptophysin levels, though not when analyzing spinophilin, synaptotagmin or staining with DiI dye (Li et al., 2016). Therefore, differences in experimental design should be considered when discussion studies that look into changes in synapse number.

Animal models of AD often recapitulate only certain aspects of the disease, based on major pathological hallmarks, such as amyloid plaques, neurofibrillary tangles, or neurodegeneration. There are however also animal models with multiple aspects of AD pathology. They can be crucial as some cellular processes might for instance be affected by both the presence of Aβ and tau. Animal models allow for correlation between changes in protein expression, different numbers of synapses, and most importantly cognitive functioning. In addition, we have discussed data from both human and rodent studies. Gene expression profile studies have shown distinct differences between human and mouse astrocytes (Zhang et al., 2016). New and better human AD models are being developed. For instance, the use of human iPSCs derived from AD patients or modified with AD risk genes will have a big impact in studying cellular processes in AD (Essayan-Perez et al., 2019; Penney et al., 2020). Development of more complex human AD models, such as for instance AD cerebral organoids could help to further understand more complex processes involving multiple cell types and interactions.

Besides synapse formation and elimination, astrocytes may also affect synapse function. Although this was out of scope for our review, our search did result in many studies reporting on how AD astrocytes affect synapse function. For instance, upregulation of cancerous inhibitor of PP2A (CIP2A) expression in AD astrocytes is linked to impaired long-term potentiation (Shentu et al., 2019). As mentioned, calcium signaling in astrocytes is closely connected to synapse functioning, and neuronal activity close to Aβ plaques is enhanced, with an increased frequency of spontaneous Ca^2+^ transients (Busche et al., 2008). There are numerous ways in which astrocytes affect Ca^2+^ signaling, for instance by affecting the release of gliotransmitters such as glutamate, GABA, ATP, and D-serine (Perea et al., 2009; Henneberger et al., 2010; Bazargani and Attwell, 2016). Furthermore, other studies show Aβ-induced impairments in synaptic potentiation of both inhibitory and excitatory synapses, leading to hyperactivity of neurons and consequential neuronal loss. These impairments are linked to excessive perisynaptic accumulation of glutamate due to disturbances in glutamate reuptake by astrocytes (Zott et al., 2019). Overall, besides the effect of astrocytes on synapse number, the effect on synapse functioning is another important element to consider in AD-related cognitive decline.

### Conclusion and Future Perspective

We conclude that AD astrocytes are involved in causing synapse loss in AD. Although this might suggest that the presence of astrocytes in AD is harmful and that removing them could have therapeutic effects, we have to be careful. For example, when pharmacologically ablating astrocytes in organotypic brain slices from 5xFAD mice, Aβ levels increased and spine density and size were reduced (Davis et al., 2021). A better treatment option might be to specifically target reactive astrocytes. Preventing or reversing AD induced astrocyte reactivity might maintain a healthy balance between synapse formation and elimination, and could therefore attenuate AD induced synapse loss and consequential cognitive decline. Several ways have been described to attenuate astrocyte reactivity (Pekny and Pekna, 2014; Smit et al., 2021); inhibition of the Janus kinase/signal transducer and activator of transcription 3 (Jak/STAT3) pathway (Ceyzériat et al., 2018), knocking out intermediate filament proteins, such as GFAP and vimentin (Kraft et al., 2013; Kamphuis et al., 2015), or connexin-43 (Ren et al., 2018), or overexpression of clusterin in astrocytes (Wojtas et al., 2020). By combining these treatments with AD models and investigating synapse number we could assess whether tempering astrocyte reactivity prevents or reduces synapse loss. This will provide important insight into new targets to attenuate cognitive decline in AD.

## Data Availability Statement

The original contributions presented in the study are included in the article/supplementary material, further inquiries can be directed to the corresponding author.

## Author Contributions

LH contributed to conceptualization, paper selection and reading, visualization, and writing of the draft. DV contributed to paper selection. EH and JM contributed to review and editing, supervision, and funding acquisition. All authors contributed to the article and approved the submitted version.

## Funding

The work described in this paper was supported by the Alzheimer Society in the Netherlands (Alzheimer Nederland WE.03-2017-04—LH and EH) and by ZonMw Memorable (733050816—EH and JM) and (733050504—JM).

## Conflict of Interest

The authors declare that the research was conducted in the absence of any commercial or financial relationships that could be construed as a potential conflict of interest.

## Publisher's Note

All claims expressed in this article are solely those of the authors and do not necessarily represent those of their affiliated organizations, or those of the publisher, the editors and the reviewers. Any product that may be evaluated in this article, or claim that may be made by its manufacturer, is not guaranteed or endorsed by the publisher.

## References

[B1] Adams J. C. (2001). Thrombospondins: multifunctional regulators of cell interactions. Annu. Rev. Cell Dev. Biol. 17, 25–51. 10.1146/annurev.cellbio.17.1.25 11687483

[B2] Allen N. J. Eroglu C. (2017). Cell biology of astrocyte-synapse interactions. Neuron 96, 697–708. 10.1016/j.neuron.2017.09.056 29096081 PMC5687890

[B3] Alonso A. C. Zaidi T. Grundke-Iqbal I. Iqbal K. (1994). Role of abnormally phosphorylated tau in the breakdown of microtubules in Alzheimer disease. Proc. Nat. Acad. Sci. U.S.A. 91, 5562–5566. 10.1073/pnas.91.12.5562 8202528 PMC44036

[B4] Bachstetter A. D. Norris C. M. Sompol P. Wilcock D. M. Goulding D. Neltner J. H. . (2012). Early stage drug treatment that normalizes proinflammatory cytokine production attenuates synaptic dysfunction in a mouse model that exhibits age-dependent progression of Alzheimer's disease-related pathology. J. Neurosci. 32, 10201–10210. 10.1523/JNEUROSCI.1496-12.2012 22836255 PMC3419360

[B5] Balu D. T. Pantazopoulos H. Huang C. C. Y. Muszynski K. Harvey T. L. Uno Y. . (2019). Neurotoxic astrocytes express the d-serine synthesizing enzyme, serine racemase, in Alzheimer's disease. Neurobiol. Dis. 130, 104511. 10.1016/j.nbd.2019.104511 31212068 PMC6689433

[B6] Bazargani N. Attwell D. (2016). Astrocyte calcium signaling: the third wave. Nat. Neurosci. 19, 182–189. 10.1038/nn.4201 26814587

[B7] Bicca M. A. Figueiredo C. P. Piermartiri T. C. Meotti F. C. Bouzon Z. L. Tasca C. I. . (2011). The selective and competitive N-methyl-D-aspartate receptor antagonist, (–)-6-phosphonomethyl-deca-hydroisoquinoline-3-carboxylic acid, prevents synaptic toxicity induced by amyloid-β in mice. Neuroscience 192, 631–641. 10.1016/j.neuroscience.2011.06.038 21756976

[B8] Blin J. Baron J. C. Dubois B. Crouzel C. Fiorelli M. Attar-Lévy D. . (1993). Loss of brain 5-HT _2_ receptors in Alzheimer's disease: *in vivo* assessment with positron emission tomography and (^18^)setoperone. Brain 116, 497–510. 10.1093/brain/116.3.497 8513389

[B9] Boisvert M. M. Erikson G. A. Shokhirev M. N. Allen N. J. (2018). The aging astrocyte transcriptome from multiple regions of the mouse brain. Cell Rep. 22, 269–285. 10.1016/j.celrep.2017.12.039 29298427 PMC5783200

[B10] Brun A. Liu X. Erikson C. (1995). Synapse loss and gliosis in the molecular layer of the cerebral cortex in Alzheimer's disease and in frontal lobe degeneration. Neurodegeneration 4, 171–177. 10.1006/neur.1995.0021 7583681

[B11] Busche M. A. Eichhoff G. Adelsberger H. Abramowski D. Wiederhold K.-H. Haass C. . (2008). Clusters of hyperactive neurons near amyloid plaques in a mouse model of Alzheimer's disease. Science 321, 1686–1689. 10.1126/science.1162844 18802001

[B12] Cacquevel M. Aeschbach L. Houacine J. Fraering P. C. (2012). Alzheimer's disease-linked mutations in presenilin-1 result in a drastic loss of activity in purified γ-secretase complexes. PLoS ONE 7, e35133. 10.1371/journal.pone.0035133 22529981 PMC3329438

[B13] Ceyzériat K. Ben Haim L. Denizot A. Pommier D. Matos M. Guillemaud O. . (2018). Modulation of astrocyte reactivity improves functional deficits in mouse models of Alzheimer's disease. Acta Neuropathol. Commun. 6, 104. 10.1186/s40478-018-0606-1 30322407 PMC6190663

[B14] Chen Y. Strickland M. R. Soranno A. Holtzman D. M. (2021). Apolipoprotein E: structural insights and links to Alzheimer disease pathogenesis. Neuron 109, 205–221. 10.1016/j.neuron.2020.10.008 33176118 PMC7931158

[B15] Choi M. Lee S.-M. Kim D. Im H.-I. Kim H.-S. Jeong Y. H. (2021). Disruption of the astrocyte–neuron interaction is responsible for the impairments in learning and memory in 5XFAD mice: an Alzheimer's disease animal model. Mol. Brain 14, 111. 10.1186/s13041-021-00823-5 34246283 PMC8272251

[B16] Christopherson K. S. Ullian E. M. Stokes C. C. A. Mullowney C. E. Hell J. W. Agah A. . (2005). Thrombospondins are astrocyte-secreted proteins that promote CNS synaptogenesis. Cell 120, 421–433. 10.1016/j.cell.2004.12.020 15707899

[B17] Chung W.-S. Clarke L. E. Wang G. X. Stafford B. K. Sher A. Chakraborty C. . (2013). Astrocytes mediate synapse elimination through MEGF10 and MERTK pathways. Nature 504, 394–400. 10.1038/nature12776 24270812 PMC3969024

[B18] Chung W.-S. Verghese P. B. Chakraborty C. Joung J. Hyman B. T. Ulrich J. D. . (2016). Novel allele-dependent role for APOE in controlling the rate of synapse pruning by astrocytes. Proc. Nat. Acad. Sci. U.S.A. 113, 10186–10191. 10.1073/pnas.1609896113 27559087 PMC5018780

[B19] Citron M. Eckman C. B. Diehl T. S. Corcoran C. Ostaszewski B. L. Xia W. . (1998). Additive effects of PS1 and APP mutations on secretion of the 42-residue amyloid beta-protein. Neurobiol. Dis. 5, 107–16. 9746908 10.1006/nbdi.1998.0183

[B20] Conti F. DeBiasi S. Minelli A. Melone M. (1996). Expression of NR1 and NR2A/B subunits of the NMDA receptor in cortical astrocytes. Glia 17, 254–258. 10.1002/(SICI)1098-1136(199607)17:3<254::AID-GLIA7>3.0.CO;2-0 8840166

[B21] Corder E. H. Saunders A. M. Strittmatter W. J. Schmechel D. E. Gaskell P. C. Small G. W. . (1993). Gene dose of apolipoprotein E type 4 allele and the risk of Alzheimer's disease in late onset families. Science 261, 921–923. 10.1126/science.8346443 8346443

[B22] Davies C. A. Mann D. M. A. Sumpter P. Q. Yates P. O. (1987). A quantitative morphometric analysis of the neuronal and synaptic content of the frontal and temporal cortex in patients with Alzheimer's disease. J. Neurol. Sci. 78, 151–164. 10.1016/0022-510X(87)90057-8 3572454

[B23] Davis N. Mota B. C. Stead L. Palmer E. O. C. Lombardero L. Rodríguez-Puertas R. . (2021). Pharmacological ablation of astrocytes reduces Aβ degradation and synaptic connectivity in an ex vivo model of Alzheimer's disease. J. Neuroinflamm. 18, 73. 10.1186/s12974-021-02117-y 33731156 PMC7972219

[B24] de Pins B. Cifuentes-Díaz C. Thamila Farah A. López-Molina L. Montalban E. Sancho-Balsells A. . (2019). Conditional BDNF delivery from astrocytes rescues memory deficits, spine density and synaptic properties in the 5xFAD mouse model of Alzheimer disease. J. Neurosci. 2121–18. 10.1523/JNEUROSCI.2121-18.2019 30700530 PMC6435824

[B25] Dekens D. W. Naudé P. J. W. Engelborghs S. Vermeiren Y. Van Dam D. Oude Voshaar R. C. . (2017). Neutrophil gelatinase-associated lipocalin and its receptors in Alzheimer's disease (AD) brain regions: differential findings in AD with and without depression. J. Alzheimer's Dis. 55, 763–776. 10.3233/JAD-160330 27716662 PMC5147520

[B26] DeKosky S. T. Scheff S. W. (1990). Synapse loss in frontal cortex biopsies in Alzheimer's disease: correlation with cognitive severity. Ann. Neurol. 27, 457–464. 10.1002/ana.410270502 2360787

[B27] Delekate A. Füchtemeier M. Schumacher T. Ulbrich C. Foddis M. Petzold G. C. (2014). Metabotropic P2Y1 receptor signalling mediates astrocytic hyperactivity *in vivo* in an Alzheimer's disease mouse model. Nat. Commun. 5, 5422. 10.1038/ncomms6422 25406732

[B28] Du S. Jin F. Maneix L. Gedam M. Xu Y. Catic A. . (2021). FoxO3 deficiency in cortical astrocytes leads to impaired lipid metabolism and aggravated amyloid pathology. Aging Cell 20, e13432. 10.1111/acel.13432 34247441 PMC8373366

[B29] Du Z. Song Y. Chen X. Zhang W. Zhang G. Li H. . (2021). Knockdown of astrocytic Grin2a aggravates β-amyloid-induced memory and cognitive deficits through regulating nerve growth factor. Aging Cell 20, e13437. 10.1111/acel.13437 34291567 PMC8373273

[B30] Eng L. F. Ghirnikar R. S. (1994). GFAP and astrogliosis. Brain Pathol. 4, 229–237. 10.1111/j.1750-3639.1994.tb00838.x 7952264

[B31] Eng L. F. vanderhaeghen J. J. Bignami A. Gerstl B. (1971). An acidic protein isolated from fibrous astrocytes. Brain Res. 28, 351–354. 5113526 10.1016/0006-8993(71)90668-8

[B32] Escartin C. Galea E. Lakatos A. O'Callaghan J. P. Petzold G. C. Serrano-Pozo A. . (2021). Reactive astrocyte nomenclature, definitions, and future directions. Nat. Neurosci. 24, 312–325. 10.1038/s41593-020-00783-4 33589835 PMC8007081

[B33] Essayan-Perez S. Zhou B. Nabet A. M. Wernig M. Huang Y.-W. A. (2019). Modeling Alzheimer's disease with human iPS cells: advancements, lessons, and applications. Neurobiol. Dis. 130, 104503. 10.1016/j.nbd.2019.104503 31202913 PMC6689423

[B34] Galloway D. A. Phillips A. E. M. Owen D. R. J. Moore C. S. (2019). Phagocytosis in the brain: homeostasis and disease. Front. Immunol. 10, 790. 10.3389/fimmu.2019.00790 31040847 PMC6477030

[B35] Gomez-Arboledas A. Davila J. C. Sanchez-Mejias E. Navarro V. Nuñez-Diaz C. Sanchez-Varo R. . (2017). Phagocytic clearance of presynaptic dystrophies by reactive astrocytes in Alzheimer's disease. Glia 66, 637–653. 10.1002/glia.23270 29178139 PMC5814816

[B36] Guerra-Gomes S. Sousa N. Pinto L. Oliveira J. F. (2018). Functional roles of astrocyte calcium elevations: from synapses to behavior. Front. Cell. Neurosci. 11, 427. 10.3389/fncel.2017.00427 29386997 PMC5776095

[B37] Haass C. Selkoe D. J. (1993). Cellular processing of beta-amyloid precursor protein and the genesis of amyloid beta-peptide. Cell 75, 1039–1042. 8261505 10.1016/0092-8674(93)90312-e

[B38] Hefendehl J. K. LeDue J. Ko R. W. Y. Mahler J. Murphy T. H. MacVicar B. A. (2016). Mapping synaptic glutamate transporter dysfunction *in vivo* to regions surrounding Aβ plaques by iGluSnFR two-photon imaging. Nat. Commun. 7, 13441. 10.1038/ncomms13441 27834383 PMC5114608

[B39] Henneberger C. Papouin T. Oliet S. H. R. Rusakov D. A. (2010). Long-term potentiation depends on release of d-serine from astrocytes. Nature 463, 232–236. 10.1038/nature08673 20075918 PMC2807667

[B40] Hillen A. E. J. Burbach J. P. H. Hol E. M. (2018). Cell adhesion and matricellular support by astrocytes of the tripartite synapse. Progress Neurobiol. 165–167, 66–86. 10.1016/j.pneurobio.2018.02.002 29444459

[B41] Hong H.-S. Hwang J.-Y. Son S.-M. Kim Y.-H. Moon M. Mook-Jung I. (2010). FK506 reduces amyloid plaque burden and induces MMP-9 in AβPP/PS1 double transgenic mice. J. Alzheimer's Dis. 22, 97–105. 10.3233/JAD-2010-100261 20847451

[B42] Hong S. Beja-Glasser V. F. Nfonoyim B. M. Frouin A. Li S. Ramakrishnan S. . (2016). Complement and microglia mediate early synapse loss in Alzheimer mouse models. Science 352, 712–716. 10.1126/science.aad8373 27033548 PMC5094372

[B43] Hongpaisan J. Sun M.-K. Alkon D. L. (2011). PKC activation prevents synaptic loss, a elevation, and cognitive deficits in Alzheimer's disease transgenic mice. J. Neurosci. 31, 630–643. 10.1523/JNEUROSCI.5209-10.2011 21228172 PMC6623433

[B44] Huang Y.-W. A. Zhou B. Nabet A. M. Wernig M. Südhof T. C. (2019). Differential signaling mediated by ApoE2, ApoE3, and ApoE4 in human neurons parallels Alzheimer's disease risk. J. Neurosci. 39, 7408–7427. 10.1523/JNEUROSCI.2994-18.2019 31331998 PMC6759032

[B45] Huang Y.-W. A. Zhou B. Wernig M. Südhof T. C. (2017). ApoE2, ApoE3, and ApoE4 differentially stimulate APP transcription and Aβ secretion. Cell 168, 427–441.e21. 10.1016/j.cell.2016.12.044 28111074 PMC5310835

[B46] Iqbal K. del C. Alonso A. Chen S. Chohan M. O. El-Akkad E. . (2005). Tau pathology in Alzheimer disease and other tauopathies. Biochim. Biophys. Acta Mol. Basis Dis. 1739, 198–210. 10.1016/j.bbadis.2004.09.008 15615638

[B47] Jacob C. P. Koutsilieri E. Bartl J. Neuen-Jacob E. Arzberger T. Zander N. . (2007). Alterations in expression of glutamatergic transporters and receptors in sporadic Alzheimer's disease. J. Alzheimer's Dis. 11, 97–116. 10.3233/JAD-2007-11113 17361039

[B48] Jain S. Yoon S. Y. Leung L. Knoferle J. Huang Y. (2013). Cellular source-specific effects of apolipoprotein (Apo) E4 on dendrite arborization and dendritic spine development. PLoS ONE 8, e59478. 10.1371/journal.pone.0059478 23527202 PMC3602301

[B49] Jansen I. E. Savage J. E. Watanabe K. Bryois J. Williams D. M. Steinberg S. . (2019). Genome-wide meta-analysis identifies new loci and functional pathways influencing Alzheimer's disease risk. Nat. Genet. 51, 404–413. 10.1038/s41588-018-0311-9 30617256 PMC6836675

[B50] Jung E. S. An K. Seok Hong H. Kim J.-H. Mook-Jung I. (2012). Astrocyte-originated ATP protects A 1-42-induced impairment of synaptic plasticity. J. Neurosci. 32, 3081–3087. 10.1523/JNEUROSCI.6357-11.2012 22378880 PMC6622014

[B51] Kamphuis W. Kooijman L. Orre M. Stassen O. Pekny M. Hol E. M. (2015). GFAP and vimentin deficiency alters gene expression in astrocytes and microglia in wild-type mice and changes the transcriptional response of reactive glia in mouse model for Alzheimer's disease: GFAP and vimentin in Alzheimer's disease. Glia 63, 1036–1056. 10.1002/glia.22800 25731615

[B52] Kobayashi E. Nakano M. Kubota K. Himuro N. Mizoguchi S. Chikenji T. . (2018). Activated forms of astrocytes with higher GLT-1 expression are associated with cognitive normal subjects with Alzheimer pathology in human brain. Sci. Rep. 8, 1712. 10.1038/s41598-018-19442-7 29374250 PMC5786045

[B53] Köpke E. Tung Y. C. Shaikh S. Alonso A. C. Iqbal K. Grundke-Iqbal I. (1993). Microtubule-associated protein tau. Abnormal phosphorylation of a non-paired helical filament pool in Alzheimer disease. J. Biol. Chem. 268, 24374–24384. 10.1016/S0021-9258(20)80536-5 8226987

[B54] Kraft A. W. Hu X. Yoon H. Yan P. Xiao Q. Wang Y. . (2013). Attenuating astrocyte activation accelerates plaque pathogenesis in APP/PS1 mice. FASEB J. 27, 187–198. 10.1096/fj.12-208660 23038755 PMC3528309

[B55] Krance S. H. Wu C.-Y. Zou Y. Mao H. Toufighi S. He X. . (2019). The complement cascade in Alzheimer's disease: a systematic review and meta-analysis. Mol. Psychiatry 26, 5532–5541. 10.1038/s41380-019-0536-8 31628417

[B56] Kulijewicz-Nawrot M. Syková E. Chvátal A. Verkhratsky A. Rodríguez J. J. (2013). Astrocytes and glutamate homoeostasis in Alzheimer's disease: a decrease in glutamine synthetase, but not in glutamate transporter-1, in the prefrontal cortex. ASN Neuro 5, AN20130017. 10.1042/AN20130017 24059854 PMC3791522

[B57] Lambert J.-C. Ibrahim-Verbaas C. A. Harold D. Naj A. C. Sims R. Bellenguez C. . (2013). Meta-analysis of 74,046 individuals identifies 11 new susceptibility loci for Alzheimer's disease. Nat. Genet. 45, 1452–1458. 10.1038/ng.2802 24162737 PMC3896259

[B58] Lee J.-H. Kim J. Noh S. Lee H. Lee S. Y. Mun J. Y. . (2021). Astrocytes phagocytose adult hippocampal synapses for circuit homeostasis. Nature 590, 612–617. 10.1038/s41586-020-03060-3 33361813

[B59] Lee K.-I. Lee H.-T. Lin H.-C. Tsay H.-J. Tsai F.-C. Shyue S.-K. . (2016). Role of transient receptor potential ankyrin 1 channels in Alzheimer's disease. J. Neuroinflamm. 13, 92. 10.1186/s12974-016-0557-z 27121378 PMC4847235

[B60] Li S. Uno Y. Rudolph U. Cobb J. Liu J. Anderson T. . (2018). Astrocytes in primary cultures express serine racemase, synthesize d -serine and acquire A1 reactive astrocyte features. Biochem. Pharmacol. 151, 245–251. 10.1016/j.bcp.2017.12.023 29305854 PMC5899945

[B61] Li Y. Chang L. Song Y. Gao X. Roselli F. Liu J. . (2016). Astrocytic GluN2A and GluN2B oppose the synaptotoxic effects of amyloid-β1-40 in hippocampal cells. J. Alzheimer's Dis. 54, 135–148. 10.3233/JAD-160297 27497478

[B62] Lichtman J. W. Colman H. (2000). Synapse elimination and indelible memory. Neuron 25, 269–278. 10.1016/S0896-6273(00)80893-4 10719884

[B63] Liddelow S. A. Guttenplan K. A. Clarke L. E. Bennett F. C. Bohlen C. J. Schirmer L. . (2017). Neurotoxic reactive astrocytes are induced by activated microglia. Nature 541, 481–487. 10.1038/nature21029 28099414 PMC5404890

[B64] Lin Y.-T. Seo J. Gao F. Feldman H. M. Wen H.-L. Penney J. . (2018). APOE4 causes widespread molecular and cellular alterations associated with Alzheimer's disease phenotypes in human iPSC-derived brain cell types. Neuron 98, 1141–1154.e7. 10.1016/j.neuron.2018.05.008 29861287 PMC6023751

[B65] Lorke D. E. Lu G. Cho E. Yew D. T. (2006). Serotonin 5-HT2A and 5-HT6 receptors in the prefrontal cortex of Alzheimer and normal aging patients. BMC Neurosci. 7, 36. 10.1186/1471-2202-7-36 16640790 PMC1523198

[B66] Luchena C. Zuazo-Ibarra J. Alberdi E. Matute C. Capetillo-Zarate E. (2018). Contribution of neurons and glial cells to complement-mediated synapse removal during development, aging and in Alzheimer's Disease. Mediators Inflamm. 2018, 1–12. 10.1155/2018/2530414 30533998 PMC6252206

[B67] Mahmoud S. Gharagozloo M. Simard C. Gris D. (2019). Astrocytes maintain glutamate homeostasis in the CNS by controlling the balance between glutamate uptake and release. Cells 8, 184. 10.3390/cells8020184 30791579 PMC6406900

[B68] Masliah E. Alford M. Mallory M. Rockenstein E. Moechars D. Van Leuven F. (2000). Abnormal glutamate transport function in mutant amyloid precursor protein transgenic mice. Exp. Neurol. 163, 381–387. 10.1006/exnr.2000.7386 10833311

[B69] Matos M. Augusto E. Agostinho P. Cunha R. A. Chen J.-F. (2013). Antagonistic interaction between adenosine A2A receptors and Na+/K+-ATPase- 2 controlling glutamate uptake in astrocytes. J. Neurosci. 33, 18492–18502. 10.1523/JNEUROSCI.1828-13.2013 24259572 PMC3834055

[B70] Matos M. Augusto E. Machado N. J. dos Santos-Rodrigues A. Cunha R. A. Agostinho P. (2012). Astrocytic adenosine A2A receptors control the amyloid-β peptide-induced decrease of glutamate uptake. J. Alzheimer's Dis. 31, 555–567. 10.3233/JAD-2012-120469 22647260

[B71] Matos M. Augusto E. Oliveira C. R. Agostinho P. (2008). Amyloid-beta peptide decreases glutamate uptake in cultured astrocytes: involvement of oxidative stress and mitogen-activated protein kinase cascades. Neuroscience 156, 898–910. 10.1016/j.neuroscience.2008.08.022 18790019

[B72] Maysinger D. Ji J. Moquin A. Hossain S. Hancock M. A. Zhang I. . (2017). Dendritic polyglycerol sulfates in the prevention of synaptic loss and mechanism of action on glia. ACS Chem. Neurosci. 9, 260–271. 10.1021/acschemneuro.7b00301 29078046

[B73] Mesquita S. D. Ferreira A. C. Falcao A. M. Sousa J. C. Oliveira T. G. Correia-Neves M. . (2014). Lipocalin 2 modulates the cellular response to amyloid beta. Cell Death Differentiation 21, 1588–1599. 10.1038/cdd.2014.68 24853299 PMC4158684

[B74] Minkeviciene R. Ihalainen J. Malm T. Matilainen O. Keksa-Goldsteine V. Goldsteins G. . (2008). Age-related decrease in stimulated glutamate release and vesicular glutamate transporters in APP/PS1 transgenic and wild-type mice. J. Neurochem. 105, 584–594. 10.1111/j.1471-4159.2007.05147.x 18042177

[B75] Morikawa M. Fryer J. D. Sullivan P. M. Christopher E. A. Wahrle S. E. DeMattos R. B. . (2005). Production and characterization of astrocyte-derived human apolipoprotein E isoforms from immortalized astrocytes and their interactions with amyloid-β. Neurobiol. Dis. 19, 66–76. 10.1016/j.nbd.2004.11.005 15837562

[B76] Neniskyte U. Gross C. T. (2017). Errant gardeners: glial-cell-dependent synaptic pruning and neurodevelopmental disorders. Nat. Rev. Neurosci. 18, 658–670. 10.1038/nrn.2017.110 28931944

[B77] Nishida H. Okabe S. (2007). Direct astrocytic contacts regulate local maturation of dendritic spines. J. Neurosci. 27, 331–340. 10.1523/JNEUROSCI.4466-06.2007 17215394 PMC6672072

[B78] Nizami S. Hall-Roberts H. Warrier S. Cowley S. A. Di Daniel E. (2019). Microglial inflammation and phagocytosis in Alzheimer's disease: potential therapeutic targets. Br. J. Pharmacol. 176, 3515–3532. 10.1111/bph.14618 30740661 PMC6715590

[B79] Norris C. M. (2005). Calcineurin triggers reactive/inflammatory processes in astrocytes and is upregulated in aging and Alzheimer's models. J. Neurosci. 25, 4649–4658. 10.1523/JNEUROSCI.0365-05.2005 15872113 PMC1201418

[B80] Nwabuisi-Heath E. Rebeck G. W. LaDu M. J. Yu C. (2014). ApoE4 delays dendritic spine formation during neuron development and accelerates loss of mature spines *in vitro*. ASN Neuro 6, AN20130043. 10.1042/AN20130043 24328732 PMC3891498

[B81] Oberheim N. A. Takano T. Han X. He W. Lin J. H. C. Wang F. . (2009). Uniquely hominid features of adult human astrocytes. J. Neurosci. 29, 3276–3287. 10.1523/JNEUROSCI.4707-08.2009 19279265 PMC2819812

[B82] Oberheim N. A. Wang X. Goldman S. Nedergaard M. (2006). Astrocytic complexity distinguishes the human brain. Trends Neurosci. 29, 547–553. 10.1016/j.tins.2006.08.004 16938356

[B83] Oddo S. Caccamo A. Shepherd J. D. Murphy M. P. Golde T. E. Kayed R. . (2003). Triple-transgenic model of Alzheimer's disease with plaques and tangles: intracellular Aβ and synaptic dysfunction. Neuron 39, 409–421. 10.1016/s0896-6273(03)00434-3 12895417

[B84] Orr A. G. Hsiao E. C. Wang M. M. Ho K. Kim D. H. Wang X. . (2015). Astrocytic adenosine receptor A2A and Gs-coupled signaling regulate memory. Nat. Neurosci. 18, 423–434. 10.1038/nn.3930 25622143 PMC4340760

[B85] Orre M. Kamphuis W. Osborn L. M. Jansen A. H. P. Kooijman L. Bossers K. . (2014). Isolation of glia from Alzheimer's mice reveals inflammation and dysfunction. Neurobiol. Aging 35, 2746–2760. 10.1016/j.neurobiolaging.2014.06.004 25002035

[B86] Osborn L. M. Kamphuis W. Wadman W. J. Hol E. M. (2016). Astrogliosis: an integral player in the pathogenesis of Alzheimer's disease. Progress Neurobiol. 144, 121–141. 10.1016/j.pneurobio.2016.01.001 26797041

[B87] Page M. J. McKenzie J. E. Bossuyt P. M. Boutron I. Hoffmann T. C. Mulrow C. D. . (2021). The PRISMA 2020 statement: an updated guideline for reporting systematic reviews. PLoS Med. 18, e1003583. 10.1371/journal.pmed.1003583 33780438 PMC8007028

[B88] Paradisi S. Sacchetti B. Balduzzi M. Gaudi S. Malchiodi-Albedi F. (2004). Astrocyte modulation of *in vitro* beta-amyloid neurotoxicity. Glia 46, 252–260. 10.1002/glia.20005 15048848

[B89] Paumier A. Boisseau S. Jacquier-Sarlin M. Pernet-Gallay K. Buisson A. Albrieux M. (2021). Astrocyte-neuron interplay is critical for Alzheimer's disease pathogenesis and is rescued by TRPA1 channel blockade. Brain 145, 388–405. 10.1093/brain/awab281 34302466

[B90] Pekny M. Pekna M. (2014). Astrocyte reactivity and reactive astrogliosis: costs and benefits. Physiol. Rev. 94, 1077–1098. 10.1152/physrev.00041.2013 25287860

[B91] Penney J. Ralvenius W. T. Tsai L.-H. (2020). Modeling Alzheimer's disease with iPSC-derived brain cells. Mol. Psychiatry 25, 148–167. 10.1038/s41380-019-0468-3 31391546 PMC6906186

[B92] Perea G. Navarrete M. Araque A. (2009). Tripartite synapses: astrocytes process and control synaptic information. Trends Neurosci. 32, 421–431. 10.1016/j.tins.2009.05.001 19615761

[B93] Pereira Diniz L. Tortelli V. Matias I. Morgado J. Bérgamo Araujo A. P. Melo H. M. . (2017). Astrocyte transforming growth factor beta 1 protects synapses against Aβ oligomers in Alzheimer's disease model. J. Neurosci. 37, 6797–6809. 10.1523/JNEUROSCI.3351-16.2017 28607171 PMC6596548

[B94] Perez-Nievas B. G. Stein T. D. Tai H.-C. Dols-Icardo O. Scotton T. C. Barroeta-Espar I. . (2013). Dissecting phenotypic traits linked to human resilience to Alzheimer's pathology. Brain 136, 2510–2526. 10.1093/brain/awt171 23824488 PMC3722351

[B95] Qiao J. Wang J. Wang H. Zhang Y. Zhu S. Adilijiang A. . (2016). Regulation of astrocyte pathology by fluoxetine prevents the deterioration of Alzheimer phenotypes in an APP/PS1 mouse model: regulation of astrocyte pathology. Glia 64, 240–254. 10.1002/glia.22926 26446044

[B96] Rao K. V. R. Curtis K. M. Johnstone J. T. Norenberg M. D. (2013). Amyloid-a inhibits thrombospondin 1 release from cultured astrocytes: effects on synaptic protein expression. J. Neuropathol. Exp. Neurol. 72, 10. 10.1097/NEN.0b013e31829bd082 23860027

[B97] Reichenbach N. Delekate A. Breithausen B. Keppler K. Poll S. Schulte T. . (2018). P2Y1 receptor blockade normalizes network dysfunction and cognition in an Alzheimer's disease model. J. Exp. Med. 215, 1649–1663. 10.1084/jem.20171487 29724785 PMC5987918

[B98] Reiman E. M. Arboleda-Velasquez J. F. Quiroz Y. T. Huentelman M. J. Beach T. G. Caselli R. J. . (2020). Exceptionally low likelihood of Alzheimer's dementia in APOE2 homozygotes from a 5,000-person neuropathological study. Nat. Commun. 11, 667. 10.1038/s41467-019-14279-8 32015339 PMC6997393

[B99] Ren R. Zhang L. Wang M. (2018). Specific deletion connexin43 in astrocyte ameliorates cognitive dysfunction in APP/PS1 mice. Life Sci. 208, 175–191. 10.1016/j.lfs.2018.07.033 30031059

[B100] Ries M. Sastre M. (2016). Mechanisms of Aβ clearance and degradation by glial cells. Front. Aging Neurosci. 8, 160. 10.3389/fnagi.2016.00160 27458370 PMC4932097

[B101] Sanchez-Mico M. V. Jimenez S. Gomez-Arboledas A. Muñoz-Castro C. Romero-Molina C. Navarro V. . (2020). Amyloid-β impairs the phagocytosis of dystrophic synapses by astrocytes in Alzheimer's disease. Glia 69, 997–1011. 10.1002/glia.23943 33283891

[B102] Schafer D. P. Lehrman E. K. Stevens B. (2013). The “quad-partite” synapse: microglia-synapse interactions in the developing and mature CNS. Glia 61, 24–36. 10.1002/glia.22389 22829357 PMC4082974

[B103] Scott H. A. Gebhardt F. M. Mitrovic A. D. Vandenberg R. J. Dodd P. R. (2011). Glutamate transporter variants reduce glutamate uptake in Alzheimer's disease. Neurobiol. Aging 32, 553.e1–e11. 10.1016/j.neurobiolaging.2010.03.008 20416976

[B104] Selkoe D. J. Hardy J. (2016). The amyloid hypothesis of Alzheimer's disease at 25 years. EMBO Mol. Med. 8, 595–608. 10.15252/emmm.201606210 27025652 PMC4888851

[B105] Shentu Y.-P. Hu W.-T. Zhang Q. Huo Y. Liang J.-W. Liuyang Z.-Y. . (2019). CIP2A-promoted astrogliosis induces AD-like synaptic degeneration and cognitive deficits. Neurobiol. Aging 75, 198–208. 10.1016/j.neurobiolaging.2018.11.023 30594047 PMC6405315

[B106] Shi Q. Chowdhury S. Ma R. Le K. X. Hong S. Caldarone B. J. . (2017). Complement C3 deficiency protects against neurodegeneration in aged plaque-rich APP/PS1 mice. Sci. Transl. Med. 9, eaaf6295. 10.1126/scitranslmed.aaf6295 28566429 PMC6936623

[B107] Shi Y. Yamada K. Liddelow S. A. Smith S. T. Zhao L. Luo W. . (2017). ApoE4 markedly exacerbates tau-mediated neurodegeneration in a mouse model of tauopathy. Nature 549, 523–527. 10.1038/nature24016 28959956 PMC5641217

[B108] Smit T. Deshayes N. A. C. Borchelt D. R. Kamphuis W. Middeldorp J. Hol E. M. (2021). Reactive astrocytes as treatment targets in Alzheimer's disease—systematic review of studies using the APPswePS1dE9 mouse model. Glia 69, 1852–1881. 10.1002/glia.23981 33634529 PMC8247905

[B109] Son S. M. Nam D. W. Cha M.-Y. Kim K. H. Byun J. Ryu H. . (2015). Thrombospondin-1 prevents amyloid beta–mediated synaptic pathology in Alzheimer's disease. Neurobiol. Aging 36, 3214–3227. 10.1016/j.neurobiolaging.2015.09.005 26452999

[B110] Stevens B. Allen N. J. Vazquez L. E. Howell G. R. Christopherson K. S. Nouri N. . (2007). The classical complement cascade mediates CNS synapse elimination. Cell 131, 1164–1178. 10.1016/j.cell.2007.10.036 18083105

[B111] Strittmatter W. J. Weisgraber K. H. Huang D. Y. Dong L. M. Salvesen G. S. Pericak-Vance M. . (1993). Binding of human apolipoprotein E to synthetic amyloid beta peptide: isoform-specific effects and implications for late-onset Alzheimer disease. Proc. Nat. Acad. Sci. U.S.A. 90, 8098–8102. 10.1073/pnas.90.17.8098 8367470 PMC47295

[B112] Takahashi K. Kong Q. Lin Y. Stouffer N. Schulte D. A. Lai L. . (2015). Restored glial glutamate transporter EAAT2 function as a potential therapeutic approach for Alzheimer's disease. J. Exp. Med. 212, 319–332. 10.1084/jem.20140413 25711212 PMC4354363

[B113] Talantova M. Sanz-Blasco S. Zhang X. Xia P. Akhtar M. W. Okamoto S. . (2013). Aβ induces astrocytic glutamate release, extrasynaptic NMDA receptor activation, and synaptic loss. Proc. Nat. Acad. Sci. U.S.A. 110, E2518–27. 10.1073/pnas.1306832110 23776240 PMC3704025

[B114] Teng Z. Guo Z. Zhong J. Cheng C. Huang Z. Wu Y. . (2017). ApoE influences the blood-brain barrier through the NF-κB/MMP-9 pathway after traumatic brain injury. Sci. Rep. 7, 6649. 10.1038/s41598-017-06932-3 28751738 PMC5532277

[B115] Terry R. D. Masliah E. Salmon D. P. Butters N. DeTeresa R. Hill R. . (1991). Physical basis of cognitive alterations in alzheimer's disease: synapse loss is the major correlate of cognitive impairment. Ann. Neurol. 30, 572–580. 10.1002/ana.410300410 1789684

[B116] Verkerke M. Hol E. M. Middeldorp J. (2021). Physiological and pathological ageing of astrocytes in the human brain. Neurochem. Res. 46, 2662–2675. 10.1007/s11064-021-03256-7 33559106 PMC8437874

[B117] Wang C. Xiong M. Gratuze M. Bao X. Shi Y. Andhey P. S. . (2021). Selective removal of astrocytic APOE4 strongly protects against tau-mediated neurodegeneration and decreases synaptic phagocytosis by microglia. Neuron 109, 1657–1674.e7. 10.1016/j.neuron.2021.03.024 33831349 PMC8141024

[B118] Wightman D. P. Jansen I. E. Savage J. E. Shadrin A. A. Bahrami S. Holland D. . (2021). A genome-wide association study with 1,126,563 individuals identifies new risk loci for Alzheimer's disease. Nat. Genet. 53, 1276–1282. 10.1038/s41588-021-00921-z 34493870 PMC10243600

[B119] Wilde M. C. Overk C. R. Sijben J. W. Masliah E. (2016). Meta-analysis of synaptic pathology in Alzheimer's disease reveals selective molecular vesicular machinery vulnerability. Alzheimer's Dementia 12, 633–644. 10.1016/j.jalz.2015.12.005 26776762 PMC5058345

[B120] Wojtas A. M. Sens J. P. Kang S. S. Baker K. E. Berry T. J. Kurti A. . (2020). Astrocyte-derived clusterin suppresses amyloid formation *in vivo*. Mol. Neurodegener. 15, 71. 10.1186/s13024-020-00416-1 33246484 PMC7694353

[B121] Wu J. Bie B. Foss J. F. Naguib M. (2020). Amyloid fibril–induced astrocytic glutamate transporter disruption contributes to complement C1q-mediated microglial pruning of glutamatergic synapses. Mol. Neurobiol. 57, 2290–2300. 10.1007/s12035-020-01885-7 32008166

[B122] Wu T. Dejanovic B. Gandham V. D. Gogineni A. Edmonds R. Schauer S. . (2019). Complement C3 is activated in human AD brain and is required for neurodegeneration in mouse models of amyloidosis and tauopathy. Cell Rep. 28, 2111–2123.e6. 10.1016/j.celrep.2019.07.060 31433986

[B123] Xu Q. Bernardo A. Walker D. Kanegawa T. Mahley R. W. Huang Y. (2006). Profile and regulation of apolipoprotein E (ApoE) expression in the CNS in mice with targeting of green fluorescent protein gene to the ApoE locus. J. Neurosci. 26, 4985–4994. 10.1523/JNEUROSCI.5476-05.2006 16687490 PMC6674234

[B124] Yassine H. N. Finch C. E. (2020). APOE alleles and diet in brain aging and Alzheimer's disease. Front. Aging Neurosci. 12, 150. 10.3389/fnagi.2020.00150 32587511 PMC7297981

[B125] Zhang Y. Sloan S. A. Clarke L. E. Caneda C. Plaza C. A. Blumenthal P. D. . (2016). Purification and characterization of progenitor and mature human astrocytes reveals transcriptional and functional differences with mouse. Neuron 89, 37–53. 10.1016/j.neuron.2015.11.013 26687838 PMC4707064

[B126] Zhao J. Davis M. D. Martens Y. A. Shinohara M. Graff-Radford N. R. Younkin S. G. . (2017). APOE ε4/ε4 diminishes neurotrophic function of human iPSC-derived astrocytes. Hum. Mol. Genet. 26, 2690–2700. 10.1093/hmg/ddx155 28444230 PMC5886091

[B127] Zhao J. O'Connor T. Vassar R. (2011). The contribution of activated astrocytes to Ab production: implications for Alzheimer's disease pathogenesis. J. Neuroinflammation 8. 10.1186/1742-2094-8-150 22047170 PMC3216000

[B128] Zott B. Simon M. M. Hong W. Unger F. Chen-Engerer H.-J. Frosch M. P. . (2019). A vicious cycle of β amyloid–dependent neuronal hyperactivation. Science 365, 559–565. 10.1126/science.aay0198 31395777 PMC6690382

[B129] Zumkehr J. Rodriguez-Ortiz C. J. Cheng D. Kieu Z. Wai T. Hawkins C. . (2015). Ceftriaxone ameliorates tau pathology and cognitive decline via restoration of glial glutamate transporter in a mouse model of Alzheimer's disease. Neurobiol. Aging 36, 2260–2271. 10.1016/j.neurobiolaging.2015.04.005 25964214

